# Identification of Reliable Components in Multivariate Curve Resolution-Alternating Least Squares (MCR-ALS): a Data-Driven Approach across Metabolic Processes

**DOI:** 10.1038/srep15710

**Published:** 2015-11-04

**Authors:** Hiromi Motegi, Yuuri Tsuboi, Ayako Saga, Tomoko Kagami, Maki Inoue, Hideaki Toki, Osamu Minowa, Tetsuo Noda, Jun Kikuchi

**Affiliations:** 1 Team for Advanced Development and Evaluation of Human Disease Models, RIKEN BioResource Center, 3-1-1 Koyadai, Tsukuba, Ibaraki, 305-0074, Japan; 2 RIKEN Center for Sustainable Resource Science, 1-7-22 Suehiro-cho, Tsurumi-ku, Yokohama, Kanagawa 230-0045, Japan; 3 Department of Cell Biology, Cancer Institute, Japanese Foundation for Cancer Research (JFCR), 3-8-31 Ariake, Koto-ku, Tokyo 135-8550, Japan; 4 Graduate School of Medical Life Science, Yokohama City University, 1-7-29 Suehiro-cho, Tsurumi-ku, Yokohama, Kanagawa 230-0045, Japan; 5 Graduate School of Bioagricultural Sciences, Nagoya University, 1 Furo-cho, Chikusa-ku, Nagoya, Aichi 464-0810, Japan

## Abstract

There is an increasing need to use multivariate statistical methods for understanding biological functions, identifying the mechanisms of diseases, and exploring biomarkers. In addition to classical analyses such as hierarchical cluster analysis, principal component analysis, and partial least squares discriminant analysis, various multivariate strategies, including independent component analysis, non-negative matrix factorization, and multivariate curve resolution, have recently been proposed. However, determining the number of components is problematic. Despite the proposal of several different methods, no satisfactory approach has yet been reported. To resolve this problem, we implemented a new idea: classifying a component as “reliable” or “unreliable” based on the reproducibility of its appearance, regardless of the number of components in the calculation. Using the clustering method for classification, we applied this idea to multivariate curve resolution-alternating least squares (MCR-ALS). Comparisons between conventional and modified methods applied to proton nuclear magnetic resonance (^1^H-NMR) spectral datasets derived from known standard mixtures and biological mixtures (urine and feces of mice) revealed that more plausible results are obtained by the modified method. In particular, clusters containing little information were detected with reliability. This strategy, named “cluster-aided MCR-ALS,” will facilitate the attainment of more reliable results in the metabolomics datasets.

“Omics” technologies, including genomics, transcriptomics, proteomics, and metabolomics/metabonomics, have been developed to obtain a bird’s-eye view of the underlying molecular networks in a cell or organism that elaborately regulate its complex biological responses^1^,^2^. Comprehensive analysis such omics approach has become possible owing to the accomplishments of recent studies that provide system-level measurements for essentially all cellular components in model organisms. Environmental factors that could affect these omics variables include diet, aging, and disease, whereas genetic variation comprises differences in sex, epigenetics, and gene polymorphisms^3^,^4^. Among omics technologies, the metabolome is quick to respond to such environmental stimuli, including changes in food intake, and thus could be used to monitor the metabolic status of the individual and indicate changes in homeostasis^5^,^6^.

Nuclear magnetic resonance (NMR) is widely used to study the metabolome, and its data reproducibility is a major advantage^7^,^8^,^9^,^10^. NMR-based metabolomics studies have been performed at different institutions, and often all of the data used in a single study have been collected on an individual instrument at a single location. Cross-site analytical validity studies have been conducted, showing that interconvertibility of NMR data among different institutions is one of the great advantages of NMR-based approaches^11^. This property is essential for the clinical application of metabolomics-derived biomarker discovery assisted by multivariate statistical approaches to the analysis of NMR datasets^12^,^13^. The most widely used classical multivariate statistical methods are k-means^14^, hierarchical cluster analysis (HCA)^5^,^15^, principal component analysis (PCA)^16^, and partial least squares discriminant analysis (PLS-DA), including orthogonal partial least squares discriminant analysis (OPLS-DA)^17^. With advances in multivariate statistical techniques, various strategies have been proposed, including independent component analysis (ICA)^18^, non-negative matrix factorization (NMF)^19^, and multivariate curve resolution (MCR)^20^,^21^,^22^. The MCR method is useful for resolving spectroscopic data featuring broad macromolecular peaks^23^ and also for estimating concentrations from metabolite mixture spectra^23^.

For use of these methods, determination of the number of components is the most important task. An incorrect choice can lead to loss of information (underestimation) or the inclusion of noise components (overestimation). Many methods have been proposed for determining the number of components, including the Kaiser criterion^24^, scree test^25^, cumulative contribution rate-based method, parallel analysis^26^, Cattell−Nelson−Gorsuch (CNG) test^27^,^28^, multiple regression^28^, and cross-validation^29^,^30^. Unfortunately, the results are often not consistent among these methods. This inconsistency makes it difficult to use ICA/NMF/MCR, as using the wrong number of components in the analysis decreases the reliability of the results.

When we began analyzing mouse urinary and fecal ^1^H-NMR spectra data using multivariate curve resolution-alternating least squares (MCR-ALS), we were faced with this problem. A wide range of different “optimal” numbers of components had been estimated by eight different methods (Supplementary Table S1). We were interested in determining the effect of changing the number of components. We compared the concentration profiles of all MCR-ALS results when the number of components was changed sequentially from three to 10, and the resulting differences were small. Similar components emerged reproducibly. However, some components emerged once or only a few times (Supplementary Figure S1 for urinary data, Supplementary Figure S2 for fecal data). From this observation, we considered that this reproducibility is useful as an indicator of the reliability of a component, i.e., that a reliable component emerges reproducibly regardless of the number of components, whereas an unreliable component emerges once or just a few times. Only reliable components are considered informative. Because a reliable component is identified by repeating the MCR-ALS calculation with a changed total number of components, it is no longer necessary to determine the number of components. The release from this constraint represents a great advantage for MCR-ALS analysis.

Based on this concept, we have established a modified method for MCR-ALS, named “cluster-aided MCR-ALS.” An evaluation of the method using mouse urinary and fecal ^1^H-NMR spectral data is reported in this study.

## Results

### Concept of cluster-aided MCR-ALS

**Figure 1:**
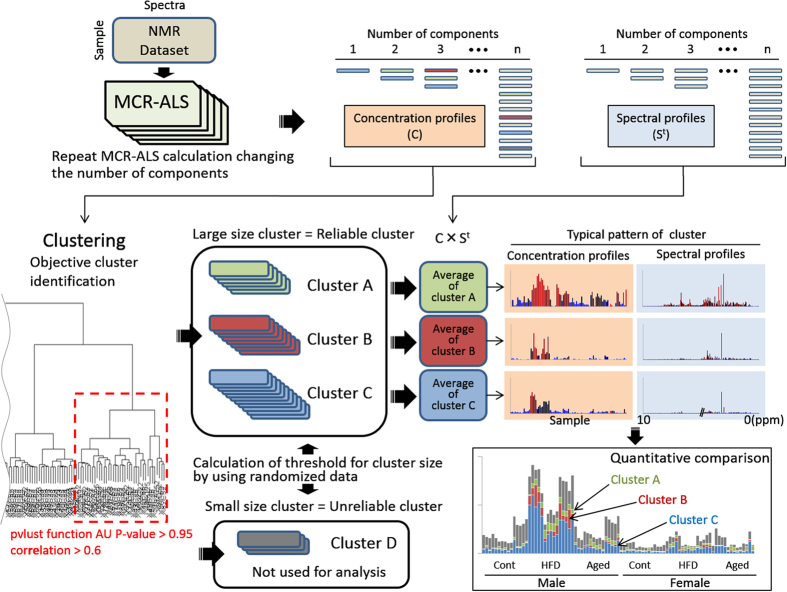
Flow chart of cluster-aided multivariate curve resolution-alternating least squares (MCR-ALS). The process of cluster-aided MCR-ALS is roughly illustrated. Details are described in the Results section.

A flow chart illustrating the process of cluster-aided MCR-ALS is shown in Fig. 1. The MCR-ALS calculation was repeated with the number of components being changed for each calculation. Numerous components were estimated, including concentration profiles and spectral profiles. All concentration profiles were collected into one dataset, and cluster analysis was performed to group those with similar patterns into single clusters. Large-sized clusters indicate that the pattern was reproduced with high frequency, regardless of the number of components. Accordingly, a large-sized cluster was considered a reliable cluster. A small-sized cluster indicated a low frequency of the emerged pattern and was considered an unreliable cluster. Accordingly, clusters of this size were not used for subsequent analysis. To perform the clustering process with objectivity, we introduced statistical cluster selection by assessing the uncertainty in hierarchical cluster analysis. For assignment of the optimum selected cluster size with objectivity, the maximum cluster size estimated from a dataset that had been randomized to destroy all biological information was set as a threshold size.

In this step, the cluster was composed on the basis only of information from the concentration profile. To integrate the information from spectral profiles, the vector product of the concentration profile (C) and the corresponding spectral profile (S^t^) was calculated (C × S^t^). Because a cluster consists of a set of matrices (C × S^t^), it is considered to be three-dimensional data (sample × spectra × elements of cluster; Supplementary Figure S3). To represent the cluster in the style of the concentration profiles and spectral profiles analogous to the conventional method, the average and the coefficient of variation (CV) of the cluster were calculated. The row and column containing the maximum values were designated as the typical spectral profile and concentration profile of the cluster, respectively (Supplementary Figure S3).

### Evaluation of the cluster-aided MCR-ALS method using known standard mixtures

To validate the methodology proposed here, known standard mixtures were introduced as model samples. Composition of the standard mixture is described in Supplementary Table S2. Varied concentrations of amino acids, short-chain fatty acids, and sugars present in urine/feces were included in this mixture.

Conventional MCR-ALS was performed for comparison with cluster-aided MCR-ALS. First, we determined the number of components using parallel analysis. Parallel analysis is similar to the simple scree test, which is widely used for PCA/factor analysis. Parallel analysis estimates the number of components in an objective manner instead of by looking for a visual leveling-off point, as in the scree test. The estimated number of components was five (Supplementary Figure S3). The resulting concentration profiles and spectral profiles are shown in Supplementary Figure S4.

Using the same dataset, cluster-aided MCR-ALS was performed. The MCR-ALS calculation was repeated, changing the number of components from one to 20. The total number of resulting components was 210. All concentration profiles were collected into one dataset on which cluster analysis was performed. To select the clusters with objectivity, we used “pvclust” developed by Suzuki *et al.*^31^. This is an R package for hierarchical clustering with probability values (*P*-values) based on a bootstrap strategy to estimate sampling error. In this study, clusters were selected by the pvclust function with an approximately unbiased (AU) *P*-value of >0.95, therefore the uncertainty of the result was less than 5%. In some cases, clusters contained undesired elements that showed little similarity to other elements within the cluster. To resolve this problem, a correlation coefficient matrix was calculated. If the minimum value was under 0.6, the cluster was rejected. Pvclust was re-executed against the rejected cluster to remove undesired elements, and the correlation coefficient was confirmed again. This process was repeated until the minimum correlation coefficient was greater than 0.6. To determine the minimum cluster size, a dataset, randomly shuffled to destroy biological information, was used. We repeated the pvclust calculation with the shuffled dataset five times. We chose the maximum cluster size for each dataset and set the threshold for the cluster size as >5. As a result, there were 15 selected clusters. Clusters and their elements (components) are listed in Supplementary Table S3. Typical concentration profiles and spectral profiles of reliable clusters are illustrated in Supplementary Figure S5.

A comparison of the results of conventional and cluster-aided MCR-ALS is summarized in supplementary Table S4. We calculated correlation coefficients between concentrations of compounds and concentration profiles estimated by MCR-ALS. Components with a correlation coefficient of >0.8 were considered as “correctly detected.” Phenylalanine (component 1), isoleucine (component 2), threonine (component 3), ethanol, sucrose (component 4), and leucine (component 5) were detected by conventional MCR-ALS. In addition to these results from the conventional method, cluster-aided MCR-ALS detected glutamate, tryptophan, proline, alanine, butyrate, glucose, and trehalose. However, aspartate, histidine, formate, citrate lactate, malate, and fructose were not detected by either conventional or cluster-aided MCR-ALS.

Two clusters were assigned to trehalose (clusters 2.3.2.4.1 and 2.3.2.4.2, see Supplementary Figure S5). In contrast, cluster 2.3.2.4.1 showed the pattern of alanine concentration and cluster 2.3.2.4.2 showed ethanol (Supplementary table S4). This result is caused by similar concentration patterns among the three compounds. Cluster-aided MCR-ALS resolved these compounds into two, and not three, clusters. Accordingly, trehalose was assigned to two clusters. This problem may be solved by increasing the number of samples, optimizing the clustering conditions, and/or increasing the resolution of binned NMR data. This comparison clearly showed that cluster-aided MCR-ALS detects more compounds than the conventional method. However, cluster-aided MCR-ALS does not detect all compounds completely, owing to resolution loss by spectral binning.

### Experimental design for sampling of mouse urine and feces

To apply the cluster-aided MCR-ALS method to biological samples, we collected ^1^H-NMR spectra data of mouse urine and feces. Because MCR-ALS can be used for multi-group analyses, we constructed a multi-group dataset. Male and female mice from three different strains were used. The mice were fed either a normal or a high-fat diet (HFD). Aged groups were also added. In total, 18 groups of NMR spectra were analyzed, concurrently, for each urine and feces sample (see Methods section).

Unlike the practice in general atherosclerosis studies, the HFD was fed to mice for only 4 days, given that metabolomic changes occur quickly in response to food changes. It was unnecessary to induce atherosclerosis in the mice, because our purpose was only to evaluate this new method. Additionally, feeding HFD for only a short period conserved both time and funds in this experiment.

### Urinary and fecal ^1^H-NMR spectra

At the outset of sample collection, 90 mice (3 strains × 2 sexes × 3 conditions × 5 mice) were available. However, one mouse, belonging to the DBA/2J female aged group, died of unknown causes. All other mice were healthy during the experimental period. Sample collection and NMR analysis were performed successfully. The final number of samples was 89.

Many urinary ^1^H-NMR spectroscopy studies performed in mice have been reported. Our urinary spectra data showed a pattern similar to that of previous reports, which include signals from acetate, citrate, creatine, creatinine, hippurate, lactate, taurine, trimethylamine (TMA), trimethylamine *N*-oxide (TMAO), and other metabolites. (Supplementary Figure S6A, Supplementary Table S5)^32^,^33^. In fecal ^1^H-NMR spectra, the peaks were broader than those in urinary spectra. To obtain more detailed information, bin width was set to 0.02 ppm, a higher resolution than that of the urinary spectra (0.04 ppm). We identified 29 metabolites in fecal extracts, including short-chain fatty acids (lactate, acetate, butanoate, and succinate) and branched-chain amino acids (leucine, isoleucine, and valine; Supplementary Figure S6B, Supplementary Table S5). These are commonly observed in feces derived from mammalian species^34^,^35^.

### Principal component analysis

To summarize the data and for comparison with MCR-ALS, PCA was performed. Details of the first four components are shown in Supplementary Figures S7 (urine) and S8 (feces). In the urinary data analysis, the normal diet group (ND; control and aged mice) and the HFD group were clearly separated in the PC1–PC2 score plot. In each group, a sex difference was also observed (Supplementary Figure S7A). A strain difference (C57BL/6J vs. others) was observed in the PC3–PC4 scores plot. However, these groups were not separated clearly (Supplementary Figure S7B). In the fecal data analysis, a strain difference was observed in the PC1–PC2 score plot (Supplementary Figure S8A). In the PC3–PC4 score plot, HFD-fed mice formed a group (Supplementary Figure S8B). However, these groups overlapped with another group. Five typical variable loadings corresponding to each group were selected, and chemical shift values were indicated (Supplementary Figures S7C, S7D, S8C, and S8D). For example, in the urinary dataset, the loading corresponding to 2.88 ppm was located in the group of HFD-fed male mice. The signal was assigned to TMA. Signals ranging from 3.5 to 4.0 ppm were located in the HFD-fed female mouse group, suggesting that sugars are related to separate in PC1 direction (Supplementary Figure S7C).

PCA is a popular multivariate analysis method. However, clustering in PCA score plot is not objective in this method, but is performed visually. Additionally, assignment of loading to the group is difficult when groups overlap. Finally, no statistical index is provided for grouping.

### Conventional MCR-ALS

**Figure 2:**
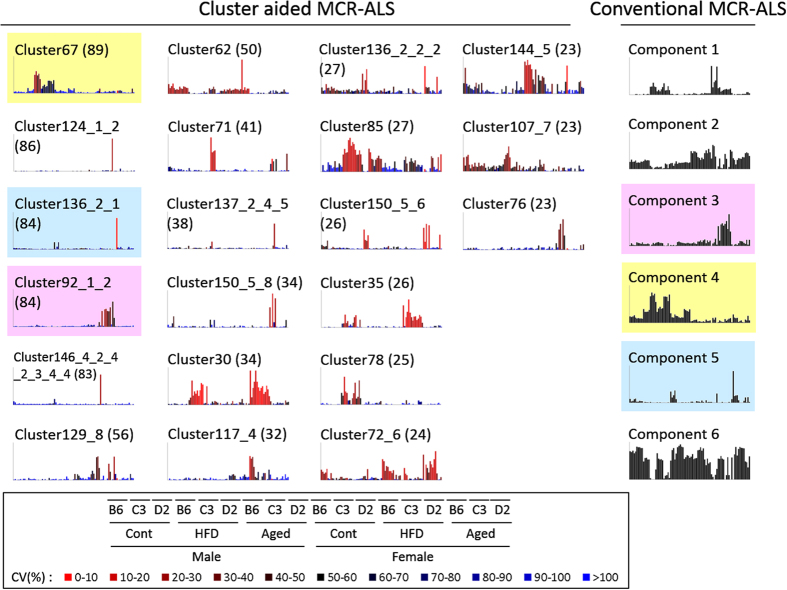
Results of cluster-aided multivariate curve resolution-alternating least squares (MCR-ALS) and conventional MCR-ALS. Concentration profiles of the results of urinary data analysis. In the bar graph, the order of the samples is indicated at the bottom of the figure. B6, C57BL/6J; C3, C3H/HeJ; D2, DBA/2J; Cont, control group; HFD, high-fat-diet-fed group; Aged, aged group. Typical concentration profiles in 21 identified reliable clusters analyzed by cluster-aided MCR-ALS are shown on the left side of the figure. Six components analyzed by conventional MCR-ALS are shown on the right side. The number in parentheses indicates the cluster size. Colored clusters/components indicate that the component belongs to the same color cluster. Scales of bar graphs are in arbitrary units. The colors of the bars correspond to coefficients of variation.

Conventional MCR-ALS was performed for comparison with cluster-aided MCR-ALS. First, we determined the number of components using parallel analysis. Parallel analysis is similar to the simple scree test, which is widely used for PCA/factor analysis. Parallel analysis estimates the number of components in an objective manner instead of by visual searching for a leveling-off point, as in the scree test. The estimated number of components was six for both urinary and fecal data (Supplementary Figure S9). The resulting concentration profiles and spectral profiles are shown in Supplementary Figures S10 (urine) and S11 (feces). Only concentration profiles of urinary data analysis are shown in the right panels of Fig. 2. In urinary data analysis, component 1 revealed a HFD-dependent increase in both sexes of all strains. Spectral profiles showed that sugar (region including 3.76 ppm), TMAO (3.26 ppm), dimethylamine (DMA; 2.71 ppm), and lactate (1.33 ppm) were associated with the change. Components 3 and 4 showed a sex-dependent increase in HFD feeding. TMAO (3.30 ppm) and TMA (2.88 ppm) contributed to the changes. In component 5, acetate (1.92 ppm) appeared to be present at high levels in aged C57BL/6J mice. A HFD-specific decrease, except in C3H/HeJ mice, was observed for component 6. Many metabolites may contribute to the decrease in component 6.

The result of fecal analysis is shown in Supplementary Figure S11. For component 4, relatively low levels were observed in C57BL/6J mice. Acetate (1.92 ppm), short-chain fatty acids (lactate, acetate, butanoate, and succinate), and branched-chain amino acids (leucine, isoleucine, and valine; region containing 0.92 ppm) may be linked to this change.

Although some strain differences were observed for components 3 and 4, no marked effect of diet or aging was observed.

### Cluster-aided MCR-ALS

The MCR-ALS calculation was repeated, changing the number of components sequentially from one to 89. The total number of resulting components was 4,005 for both the urinary and fecal datasets. The components were reduced to 3,077 (urine) and 2,550 (feces) after components with all elements zero were removed. All concentration profiles were combined into one dataset on which cluster analysis was performed. Cluster selection was performed in the same way as standard mixture analysis except that analysis of shuffled data was repeated 10 times for determining the minimum cluster size. Finally, we identified 21 (urine) and 35 (feces) reliable clusters. Clusters and their elements (components) are listed in Supplementary Tables S6 (urine) and S7 (feces). Typical concentration profiles and spectral profiles of reliable clusters are illustrated in Supplementary Figures S12 (urine) and S13 (feces).

### Comparison between conventional and cluster-aided MCR-ALS

Concentration profiles for both methods of urinary analysis are shown in Fig. 2. In cluster-aided MCR-ALS, 21 reliable clusters were estimated, a number larger than the number of components (six) in conventional MCR-ALS, estimated by parallel analysis. Three pairs (cluster 92_1_2 vs. component 3, cluster 67 vs. component 4, cluster 136_2_1 vs. component 5) were composed of a cluster and its element, indicating that a similar pattern was observed for both methods. However, components 1, 2, and 6 of conventional MCR-ALS were not elements of any of the clusters identified by cluster-aided MCR-ALS, suggesting that they were elements of unreliable clusters. For component 1, similar patterns were observed for clusters 30 and 35. It is possible that component 1 and clusters 30 and 35 did not group into a common cluster because of incomplete optimization of the clustering conditions.

Supplementary Figure S14 shows the concentration profiles from the fecal data analysis, in which 35 clusters were assigned. As with the urinary analysis, the number of reliable clusters was larger than the number of components (six) in the conventional method. Four pairs (cluster 176 vs. component 2, cluster 220_2_1 vs. component 5, cluster 189 vs. component 4, cluster 203_9 vs. component 1) were clusters and their elements. Cluster 224_10 and cluster 210_2_7 showed high CVs caused by low concentrations.

**Figure 3:**
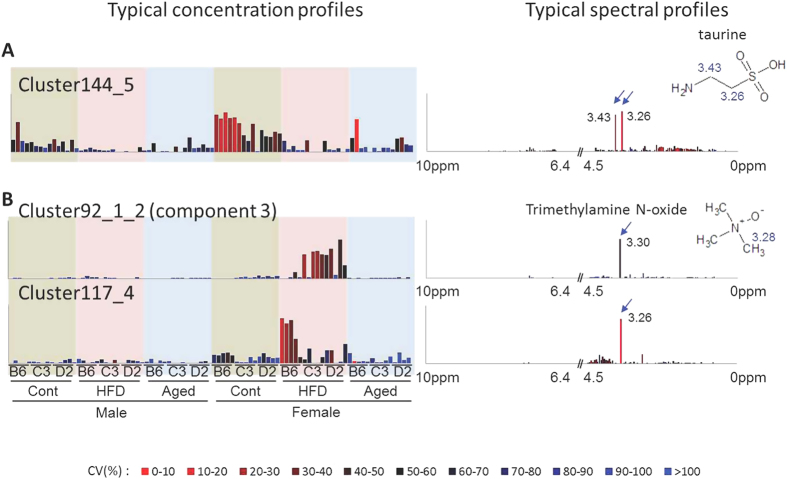
Details of the selected results of cluster-aided multivariate curve resolution-alternating least squares. (**A**) Typical concentration profile and spectral profile of cluster 144_5 in urine analysis. (**B**) Profiles of clusters 92_1_2 and 117_4 in urine analysis. The colors of the bars correspond to coefficients of variation.

For further comparison, we focused on three metabolites: taurine, TMAO, and TMA, because they are well characterized in the field of urine metabolomics. The ^1^H-NMR chemical shifts of taurine were 3.26 and 3.43 ppm. The signals were confirmed by ^1^H-^13^C-heteronuclear single quantum coherence (HSQC)-NMR. From the result of cluster-aided MCR-ALS, both taurine signals were observed in cluster 144_5 (Fig. 3A). The concentration profile showed higher levels in control mice than in HFD or aged mice. However, taurine signals were not observed in the results of conventional MCR-ALS (Supplementary Figure S10).

The TMAO signal was assigned to 3.28 ppm in ^1^H-^13^C-HSQC-NMR spectra. Unfortunately, 3.28 ppm was situated at the boundary of two bins (3.26 and 3.30 ppm). The TMAO signal was accordingly assigned as either 3.26 or 3.30 ppm because of variation in experimental conditions such as sample temperature, pH, and ionic strength. The TMAO signal was observed in cluster 92_1_2 (3.30 ppm) and cluster 117_4 (3.26 ppm; Fig. 3B). High values in HFD-feeding female mice (except in C57BL/6J) were observed in cluster 92_1_2. In contrast, only C57BL/6J female mice showed a high level in cluster 117_4. In conventional MCR-ALS, cluster 92_1_2 and component 3 showed a similar pattern, whereas no cluster 117_4-like pattern was identified.

Clusters 67, 78, and 85 showed high levels of TMA (2.88 ppm) in male HFD-fed mice (Supplementary Figure S12). In conventional MCR-ALS, cluster 67 and component 4 displayed similar patterns, whereas cluster 78- and 85-like patterns were not observed.

### Quantitative analysis

**Figure 4:**
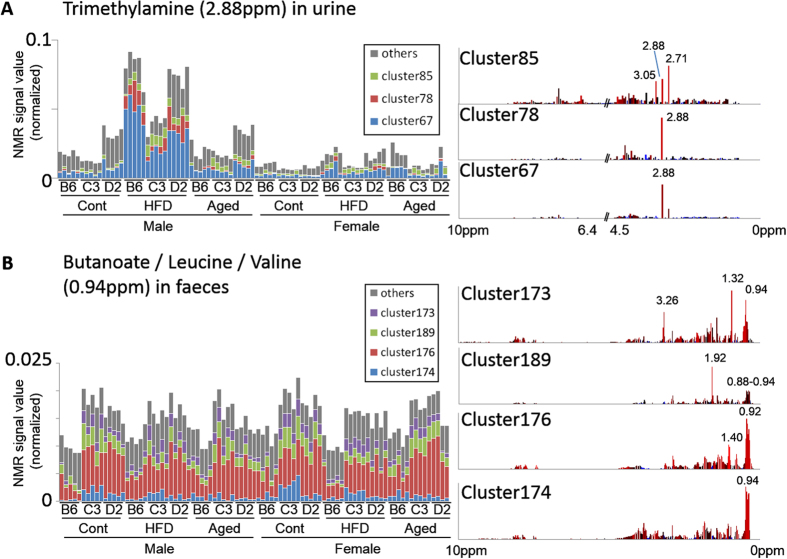
Color-coding bar graph representation. (**A**) Signals of 2.88 ppm in urine. (**B**) Signals of 0.94 ppm (feces). Right panels show spectral profiles of each cluster.

Figure 4A shows a quantitative comparison using a color-coded bar graph representation of the 2.88-ppm value corresponding to the TMA signal. This value consists mostly of clusters 67, 78, and 85. The graph shows that cluster 67 is a major contributor to the increase of TMA. Although clusters 78 and 85 are minor contributors, it appears that these clusters carry biological information. In cluster 85, a strong 2.71 ppm signal corresponding to DMA was observed. Cluster 85 may indicate a TMA- and DMA-coupled metabolic pathway. Cluster 78 may be another minor TMA-associated pathway.

The color-coded bar graph in Fig. 4B represents the 0.94 ppm signal from the fecal NMR analysis. Cluster 176 was a large contributor to this signal, showing a lower intensity level in C57BL/6J mice in all conditions. Cluster 174, with higher intensity levels in control C3H/HeJ female mice, showed a smaller contribution to the signal. The change observed in cluster 174 was not present in other clusters, therefore mainly contributed by cluster 176.

## Discussion

In PCA, the importance of a principal component is assessed by its eigenvalue or contribution. These parameters are based on the amount of information in each component; in this strategy, components with large amounts of information are preferentially chosen over components with less information. Many methods for determining numbers of components are also based on the amount of information. However, although there is no evidence to suggest that all biologically informative components contain large amounts of information; biologically informative components with small amounts of information are not detected by conventional methods. In biological systems, large changes may be triggered by small changes^5^,^34^,^36^. For example, it is probably necessary to detect small changes (with small amounts of information) for early detection of disease (thus, pre-symptomatic changes) for disease prevention. In this study, we implemented a novel idea: the classification of components as either “reliable” or “unreliable.” This classification is based on the reproducibility of similar components when the number of components in an MCR-ALS calculation is varied. Since this strategy has no relation to the amount of information, cluster-aided MCR-ALS can identify more components with low information than can the conventional method. Indeed, the numbers of estimated clusters were larger than those from the parallel analysis, which was used for conventional MCR-ALS to determine the number of components. To assess the amount of information within a cluster, the sum of squared deviations was calculated (Supplementary Figure S15).

In the urinary data analysis, clusters 67 and 92_1_2 showed a high sum of squared deviations. These patterns were observed in both cluster-aided MCR-ALS and the conventional method (red bar in Supplementary Figure S15A). Furthermore, these patterns (diet difference and sex difference) corresponded to the pattern of the PC1–PC2 score plot of PCA.

In the fecal analysis, patterns of clusters 176 and 203_9 were observed in both the cluster-aided and conventional methods (red bar in Supplementary Figure S15B). Cluster 176 showed low levels in C57BL/6J mice and cluster 203_9 shows a countertrend. This pattern corresponds to the PC1–PC2 plot of PCA. These clusters are of major components containing large amounts of information.

Other clusters showed a smaller sum of squared deviations, and most of these were identified by cluster-aided MCR-ALS. As seen in Fig. 4A, clusters 78 and 85 in the urinary analysis may contain biological information associated with TMA metabolism. Cluster 174 in the fecal analysis (Fig. 4B) may reflect an unknown metabolism associated with strain differences. Cluster-aided MCR-ALS has the potential to detect hidden biological process observed by analytical data sets.

In this study, we evaluated a cluster-aided MCR-ALS method by focusing on three metabolites: taurine, TMAO, and TMA. A decrease in mouse urinary taurine under HFD feeding conditions has been reported^37^. The results of microarray analysis indicate that transcriptional downregulation occurred in the genes of the taurine synthesis pathway, including those encoding cysteine dioxygenase (Cdo), cysteine sulfinate decarboxylase (Csd), and cystathionine beta-synthase (Cbs), in the livers of HFD-fed C57BL/6J male mice^38^. An age-associated decrease in urinary taurine in Fischer 344 male rats was reported by Dawson *et al.*^39^. Other groups have observed a similar age-related taurine decrease in the plasma and liver of rats^40^,^41^. In mice, Cbs was less efficient in the livers of aged mice^42^. Considering these results, we may infer that both HFD feeding and aging reduce urinary taurine levels. In the results of cluster-aided MCR-ALS, in both HFD feeding and aging, decreases were observed in cluster 144_5 (Fig. 3A). Cluster 144_5 may indicate agreement with the findings of previous reports on taurine metabolism. It is worth mentioning that we did not detect taurine signals in the results of the conventional analysis, suggesting that cluster-aided MCR-ALS gives more accurate results than the conventional method.

With respect to TMA, we observed high levels (2.88 ppm) in the HFD-fed group in both conventional and cluster-aided MCR-ALS. TMA is produced from choline by gut microbiota and converted to TMAO by flavin-containing monooxygenase form 3 (FMO3) in liver^43^,^44^. FMO3 activity shows a difference between the sexes in mice (with females having higher levels), whereas no difference has been observed in humans^45^. Thus, female mice typically have lower levels of TMA and higher levels of TMAO than males. Results of both the conventional and cluster-aided MCR-ALS methods showed this change (TMA = cluster67:component4, TMAO = cluster92_1_2:component3). Additionally, cluster 117_4, which had a shifted signal of TMAO in only the C57BL/6J mice, was identified by cluster-aided MCR-ALS. Binning of spectra is a commonly used method for the reduction of data size. However, in some instances, identical signals are allocated to different bins because of variation among samples. In this study, cluster-aided MCR-ALS was able to differentiate and classify variable signals successfully, whereas the same signals could not be identified by the conventional method.

In the urinary analysis, pvclust initially identified 151 clusters with an AU *P*-value of >0.95, whereas the final number of retained clusters was 21. In a similar manner, 231 clusters were identified and 35 clusters were retained in the fecal data analysis. In both datasets, approximately 85% of clusters were considered unreliable. We cannot yet explain why unreliable components comprise a large fraction of total clusters. Unreliable clusters may reflect a loss of precision caused by an excess of variables. In theory, the number of samples is larger than the number of variables. However, in many cases, this is not practical in the analysis of omics datasets. Another factor causing this is the threshold values for selecting reliable clusters. Two threshold values are important; one is the AU *P*-value, estimated by the R package pvclust. We used 0.95 as a threshold value. Higher values, such as 0.97, 0.98, or 0.99 should be tested to optimize analytical conditions. The other threshold value is the size of the cluster, which reflects the reproducibility of the component. A large size means a highly reproducible component. To determine the threshold size, pvclust was performed with a randomly shuffled dataset. A reliable cluster size should be larger than the threshold size. In principle, this process should be repeated many (at least 1,000) times. However, we repeated the analysis only 10 times (five times in standard mixture analysis) because of limited access to a high-performance computing environment. It is also necessary to optimize the clustering algorithm, which may be Euclidean, maximum, or Manhattan for the distance matrix, or methods such as Wards, single, complete, and median for cluster forming. Because this calculation must be repeated many times, high-performance computer resources such as a supercomputer should be used.

In summary, we introduced the idea of “reliable” and “unreliable” components based on the reproducibility of components in repeated MCR-ALS calculations with the number of components changed for each calculation. We evaluated this strategy, named “cluster-aided MCR-ALS,” using urinary and fecal ^1^H-NMR datasets as a test case. Concentration and spectral profiles of identified reliable clusters showed more plausible patterns than the results of conventional MCR-ALS. Cluster-aided MCR-ALS avoids the need to determine the number of components prior to the analysis, a requirement of the conventional method. This report has shown that cluster-aided MCR-ALS is a feasible method for analysis of ^1^H-NMR datasets. Cluster-aided MCR-ALS will also be applicable to other omics data. The algorithm can also be applied to other methods such as ICA/NMF. Optimization of the conditions and speed of the calculation will be necessary for further development of the cluster-aided MCR-ALS strategy.

## Methods

### Chemicals

The internal standard for ^1^H-NMR spectroscopy, 3-(trimethylsilyl)-1-propanesulfonate sodium salt (DSS), was purchased from Sigma-Aldrich Japan. Stable isotopically labeled substrate, ^13^C_6_-D-Glucose, was obtained from Cambridge Isotope Laboratories, Inc., USA.

### Animal handling

All animal experiments were approved by the Animal Research Committee of the RIKEN BioResource Center and were performed in accordance with RIKEN guidelines for animal experiments. Three laboratory mouse strains, C57BL/6J, C3H/HeJ, and DBA/2J, were purchased from a commercial breeder (CLEA Japan, Inc., Japan) and maintained in our facility (RIKEN BioResource Center Research Building for Animal Models of Human Disease). All mice were maintained at constant room temperature (23 °C ± 2 °C) and humidity (55 ± 10%) under a light/dark cycle of 12/12 h in a specific pathogen-free (SPF) environment. The pathogens were specified in categories A and B of the International Council for Laboratory Animal Science. Mice were weaned between the ages of 4 and 5 weeks. Mice had *ad libitum* access to water and conventional chow diet, CA-1 (CLEA Japan, Inc., Japan).

### Sample collection

Laboratory mice were divided into three experimental groups, “control,” “HFD feeding,” and “aged,” from which all NMR samples were collected. The control group consisted of 30 14-week-old mice, both females and males, of three strains (five mice from each sex of each strain). On day 1, the mice were placed into the metabolic cage unit (CL-0355; CLEA Japan, Inc., Japan) in the evening. Mice had access to ND, which is a conventional chow diet (CA-1) and 2% ^13^C_6_-D-glucose water, *ad libitum*. After habituation for 4 days (from the evening of day 1 to day 4), urine and feces were collected within a time span of 18 h from the evening of day 4 to the morning of day 5.

For the HFD-feeding group, 16-week-old mice were used. Except for the use of a HFD, the number of mice and sampling procedures were the same as the control group. The period of HFD feeding was approximately 4 days (evening of day 1 to morning of day 5).

Sampling was performed in the same way for the aged group as for the control group. However, for the aged group, 60-week-old mice were used for urine and feces collection.

The HFD used in this study was custom-designed based on CA-1 feed (CLEA Japan, Inc., Japan), and named HFD-RIKEN. It was composed of 70% CA-1, 18.55% granulated sugar, 10% cocoa butter, 1.25% cholesterol, and 0.2% cholate.

### Sample preparation and ^1^H-NMR spectroscopy

Urine and feces extract samples were suspended in 10% (v/v) deuterium oxide (D_2_O), and 1 mM sodium 2,2-dimethyl-2-silapentane-5-sulfonate (DSS) was used as an internal standard. After centrifugation, the extracted supernatant was transferred into a 5-mm Φ NMR tube. All one-dimensional (1D) Watergate spectra were acquired at 298 K on a DRX-500 spectrometer (Bruker Biospin, Rheinstetten, Germany), operating at 500.13 MHz and equipped with a ^1^H inverse triple-resonance probe with triple-axis gradients (Bruker Biospin), as previously described^46^. Briefly, 32,768 data points with a spectral width of 12,500 Hz were collected into 16 transients and one dummy scans, and residual water signals were suppressed by Watergate pulse sequence with a 2-s cycle time. Prior to Fourier transformation, the free induction decays were multiplied by an exponential window function corresponding to a 0.3 Hz line broadening factor. The acquired spectra were manually phased and baseline-corrected. Two-dimensional (2D) ^1^H-^13^C-HSQC) spectra were recorded on a Bruker DRU-700 NMR spectrometer equipped with a ^1^H inverse cryogenically cooled probe with a z-axis gradient as previously described^47^,^48^,^49^,^50^. All NMR spectra were processed using NMRPipe software^51^ and assigned using the SpinAssign program on the PRIMe website^52^,^53^.

### Data processing

The series of ^1^H-NMR spectra data (0–10 ppm) obtained was binned with 0.04-ppm (urine) and 0.02-ppm (feces) intervals to result in datasets of 250 (urine) and 500 (feces) variables. The region of the peak of DSS (urine: 0–0.277 ppm, feces: 0–0.48 ppm) was eliminated. For urine spectra, the region from 4.5 ppm to 6.25 ppm was excluded to eliminate the signals from both water and urea. Each spectrum was normalized to a total intensity.

### Data analysis

The MCR-ALS method was used to resolve multiple component responses in unknown mixtures^54^,^55^. The dataset (D) is expressed as a combination of concentration profiles (C) and spectral profiles (S) using the following equation:


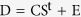


where E is a residual. Matrices C and S are estimated by an optimization algorithm based on a bilinear model with constraints of non-negativity. For analysis of MCR-ALS, the R package *ALS* was installed. Before MCR-ALS was performed, PCA was performed for estimation of the initial concentration matrix. PCA was performed using the *prcomp* R function. The PCA score (value “x” of the result of *prcomp*) was used for the initial concentration matrix. For the initial spectral matrix, all values were set to 1.

For cluster-aided MCR-ALS, the calculation for MCR-ALS was repeated, changing the number of components from one, sequentially, to the maximum number of components. Because the PCA score was used for the initial matrix, the maximum number of components was the same as the number of samples. Estimated concentration profiles were combined into one dataset (called “CList-data”) and applied to the clustering process. The R package pvclust was used for clustering^31^. Pvclust calculates *P*-values for the uncertainty of each cluster using bootstrap resampling. Pvclust was applied using the average method and a correlation-based distance matrix. The bootstrap sample number was set to 1,000. The package “snow” was used for parallel computing for high speed performance of the pvclust function. The clusters that had high AU *P*-values (>0.95) were picked using the “pvpick” function. If some of the clusters had inclusion relationships, only the largest cluster was selected (max.only = TRUE) to avoid overlap.

To determine the threshold size of the cluster, a randomly shuffled dataset, randomized-CList-data, was constructed. Because randomized-CList-data lacks biological information, clusters are formed by chance. The randomly shuffled dataset was constructed by the “sample” function of R, and pvclust and pvpick (AU >0.95) were performed. The randomized data clustering was performed 10 times for each urinary and fecal dataset. The maximum size of the cluster was used as the threshold cluster size.

Sometimes a cluster contains unrelated elements that show little similarity with other elements. To confirm the similarity among elements in the cluster, a correlation coefficient matrix was estimated. If the minimum value of the correlation coefficient was less than 0.6, this cluster was clustered again to divide it into smaller clusters, and then the correlation coefficient matrix was estimated again. This process was repeated until the minimum correlation coefficient was greater than 0.6 or the size of the cluster was below the threshold value determined by randomized data analysis. Selected clusters were considered “reliable clusters.”

Clusters were constructed solely from the information from concentration profiles. To incorporate the information from spectral profiles, the vector product of C and corresponding S^t^ was calculated for each component (C × S^t^; Supplementary Figure S3). The mean, standard deviation, and CV of the components in the cluster were calculated. Typical concentration and spectral profiles were selected in the average cluster showing the maximum value in the matrix (Supplementary Figure S3).

To perform conventional MCR-ALS for comparison with cluster-aided MCR-ALS, the number of components was estimated using eight different methods (Supplementary Table S1). (1) The Kaiser criterion is a simple method: factors with an eigenvalue greater than 1.0 are retained. The eigenvalue is calculated by PCA. (2) The scree test is a graphical strategy for determining the number of components. Eigenvalues and their component numbers are plotted. The number of components is the point reached before leveling-off of the plot. (3) Parallel analysis is a method based on the generation of random variables to determine the number of factors to retain. The function “nScree” in the R package “nFactors” was used. (4) CNG test is based on the comparison between the slope of the first three eigenvalues and the slope of the next three eigenvalues. Then the process is repeated. This test was performed using the “nCng” function in the nFactors package. (5) The multiple regression procedure is an extended CNG test. A series of two regression lines, one for the important components and the other component not necessary the scree test, are compared. The “nMreg” function in the nFactors package is used to perform the calculation. (6, 7) Cross-validation is a method for determining the number of components, performed by the “estim_ncp” function in the R package “FactoMineR.” Two methods, “general cross-validation” and “smoothing method,” are available in the estim_ncp function. (8) The contribution rate-based method is a method in which the number of components is determined when the PCA cumulative contribution rate >90%.

## Additional Information

**How to cite this article**: Motegi, H. *et al.* Identification of Reliable Components in Multivariate Curve Resolution-Alternating Least Squares (MCR-ALS): a Data-Driven Approach across Metabolic Processes. *Sci. Rep.* **5**, 15710; doi: 10.1038/srep15710 (2015).

## Supplementary Material

Supplementary Information
