# Supplementary material for: Identification of Reliable Components in Multivariate Curve Resolution-Alternating Least Squares (MCR-ALS): a Data-Driven Approach across Metabolic Processes
Source: Sci Rep. 2015 Nov 4;5:15710. doi: 10.1038/srep15710 (PMC4632111; doi:10.1038/srep15710)
Supplement: Supplementary Information [file srep15710-s1.pdf]

Supplementary Information

Identification of Reliable Components in Multivariate Curve Resolution-Alternating Least Squares (MCR-ALS): a Data-Driven Approach across Metabolic Processes

Hiromi Motegi [1], Yuuri Tsuboi [2], Ayako Saga [1], Tomoko Kagami [1], Maki Inoue[1], Hideaki Toki[1], Osamu Minowa[1], Tetsuo Noda [1,3] and Jun Kikuchi [2,4,5]*

[1]Team for Advanced Development and Evaluation of Human Disease Models, RIKEN BioResource Center, 3-1-1 Koyadai, Tsukuba, Ibaraki, 305-0074, Japan

[2] RIKEN Center for Sustainable Resource Science, 1-7-22 Suehiro-cho, Tsurumi-ku, Yokohama, Kanagawa 230-0045, Japan

[3]Department of Cell Biology, Cancer Institute, Japanese Foundation for Cancer Research (JFCR), 3-8-31 Ariake, Koto-ku, Tokyo 135-8550, Japan

[4]Graduate School of Medical Life Science, Yokohama City University, 1-7-29 Suehiro-cho, Tsurumi-ku, Yokohama, Kanagawa 230-0045, Japan

[5]Graduate School of Bioagricultural Sciences, Nagoya University, 1 Furo-cho, Chikusa-ku, Nagoya, Aichi 464-0810, Japan

Supplementary Table S1

Estimation of component number by eight different methods

Method	Urinary dataset	Fecal dataset
Kaiser criterion	19	23
Scree test	5	6
Parallel analysis	6	6
Cattell–Nelson–Gorsuch	3	3
Multiple regression	4	4
Cross-validation (generalized cross-validation approximation)	70	53
Cross-validation (smoothing method)	0	27
Contribution rate in principal component analysis (>90%)	14	12

Supplementary Figure S1

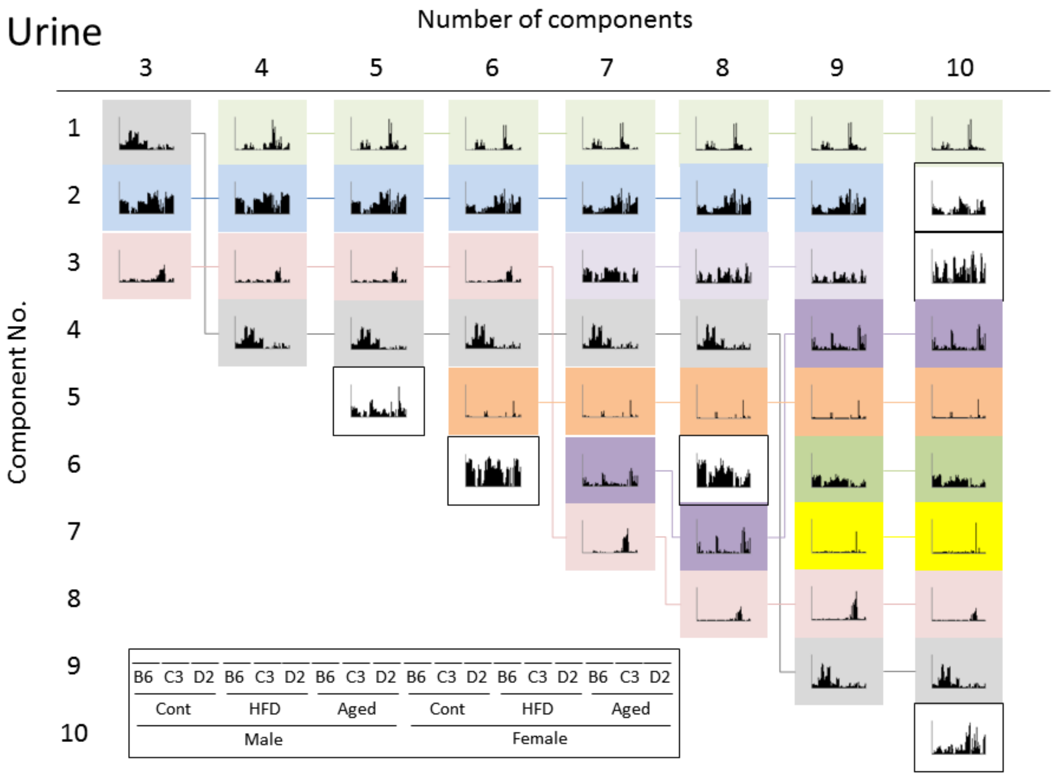

Supplementary Figure S1. The results of multivariate curve resolution-alternating least squares (MCR-ALS), changing the number of components (urinary dataset). The number of components is changed sequentially from three to 10. Resulting concentration profiles are represented longitudinally. Components that show similar patterns are painted in the same color, and linked by a line. Components not showing a similar pattern are boxed with a black line. In the bar graph, the order of the samples is indicated at the bottom left of the figure.

Supplementary Figure S2

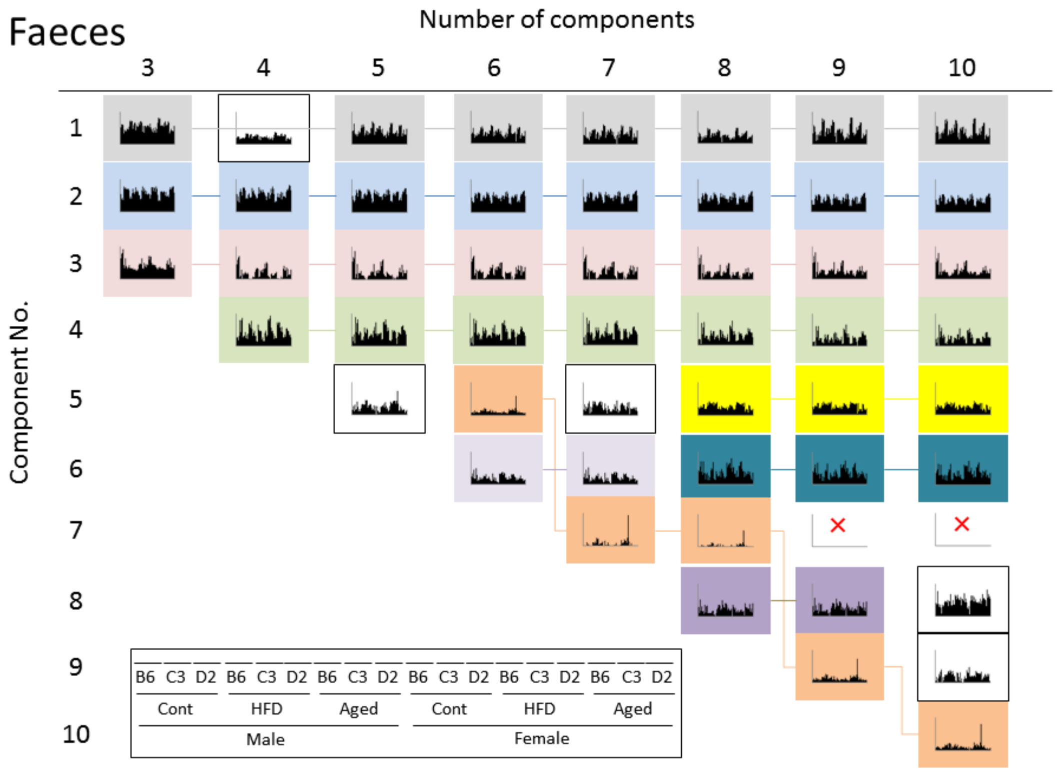

Supplementary Figure S2. Results of multivariate curve resolution-alternating least squares (MCR-ALS), changing the number of components (fecal dataset).

The number of components is changed sequentially from three to 10. Resulting concentration profiles are represented longitudinally. Components that show similar patterns are painted in the same color, and linked by a line. Components not showing a similar pattern are boxed with a black line. In the bar graph, the order of the samples is indicated at the bottom left of the figure. A red “X” mark indicates that all concentration values are zero.

Supplementary Figure S3

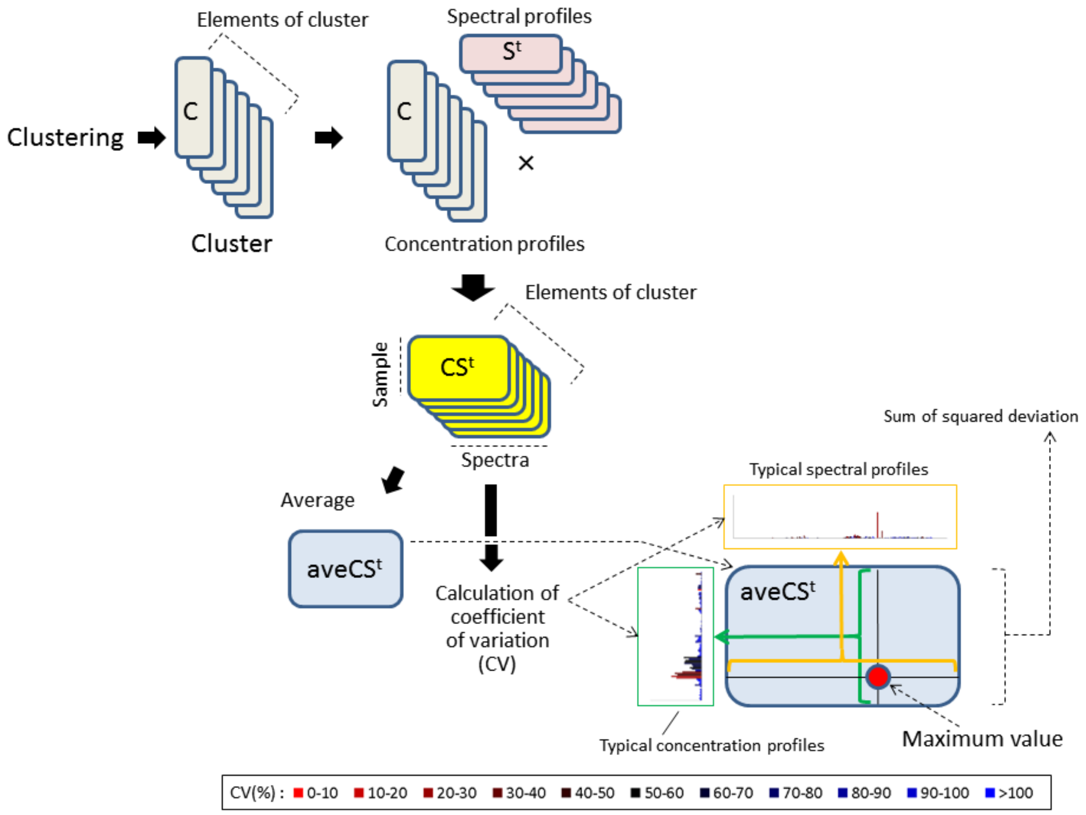

Supplementary Figure S3. Integration of spectral profiles into the clustered data matrix and extraction of typical patterns (concentration and spectral profiles) from the three-dimensional dataset.

The vector product of the concentration profile (C) and corresponding spectral profile (S^t) is calculated for each component of the individual clusters. The resulting matrix (CS^t) is a three-dimensional dataset. To reduce the dimension of the CS^t, the average is calculated. The typical pattern of the concentration and spectral profiles is then selected from the average data ($aveCS^t$). To reflect the characteristics of the cluster in the typical concentration and spectral profiles, we specify that both of these have maximum values in the $aveCS^t$ matrix. The coefficient of variation (CV) is calculated to describe deviations from the typical concentration and spectral profiles.

Supplementary Table S2

Compositions of compound mixtures

	mix20				mix15				mix10				mix5				mix3			
	1	2	3	4	1	2	3	4	1	2	3	4	1	2	3	4	1	2	3	4
Phenylalanine	1	3	5	3	1	3	5	10	1	3	5	10	1	3	5	10	3	5	10	5
Gluamate	3	1	3	1	3	1	3	1	3	5	1	3	3	5	10	1				
Leucine	1	1	3	3	10	1	3	5	10	5	1	3	5	10	1	3				
Tryptophan	3	3	1	1	3	5	3	1	1	5	3	10								
Aspartate	1	3	1	3	1	3	1	3	1	1	10	3								
Histidine	3	5	5	1	3	5	10	1												
Proline	5	10	1	3	5	10	1	3												
Alanine	10	1	3	5	10	1	3	5												
Threonine	1	3	5	3	1	3	5	10												
Isoleucine	3	5	10	1																
Butyrate	3	5	3	1	3	5	1	3	3	5	1	3	5	3	5	5	1	3	5	5
Formate	1	3	5	10	1	3	5	1	1	3	5	1								
Citrate	5	10	1	3	5	1	3	5	5	1	3	5								
Lactate	5	1	3	5					5	1	3	5								
Ethanol	5	3	1	3																
Malate	3	1	3	5																
Glucose	1	3	5	10	1	3	5	1	1	3	5	1	10	5	3	5	10	5	3	5
Trehalose	10	1	3	5	5	1	3	5												
Fructose	3	5	3	1	3	5	1	3												
Sucrose	5	5	1	3																

(mM)

Supplementary Figure S3

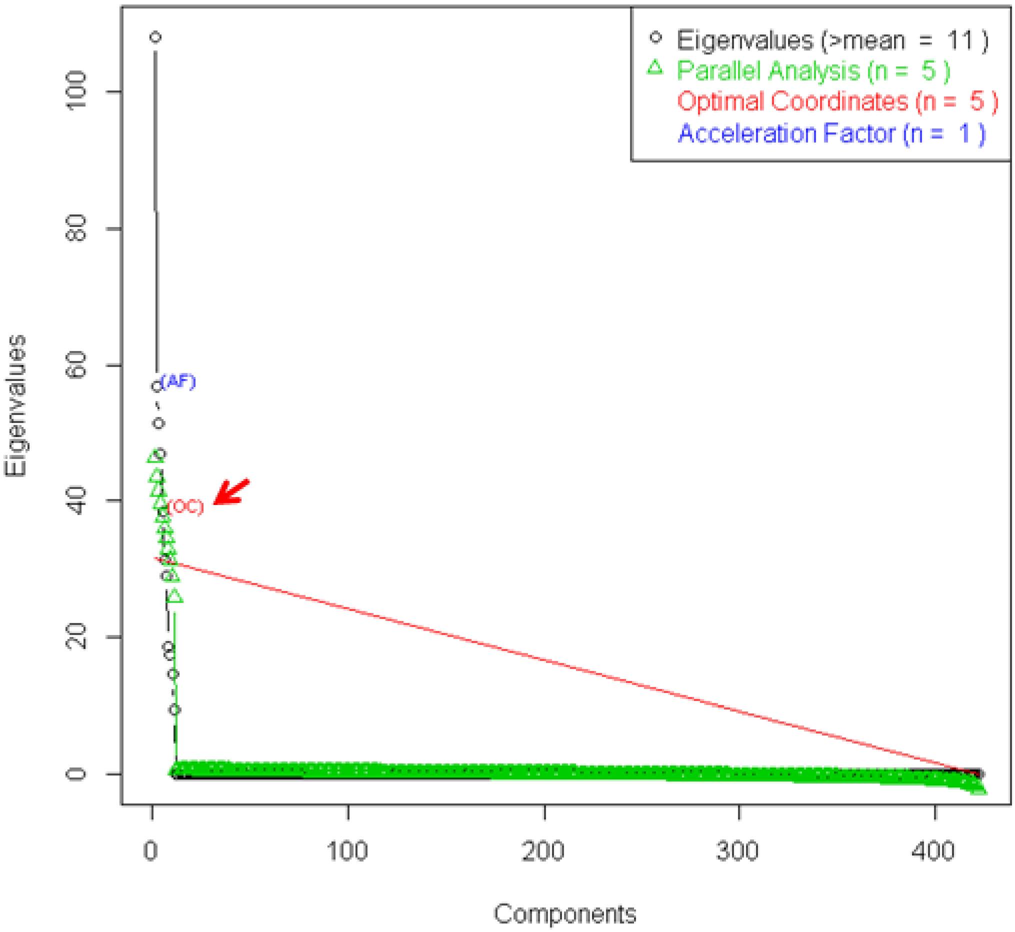

Supplementary Figure S3 Determination of the number of components for conventional multivariate curve resolution-alternating least squares (MCR-ALS) by parallel analysis.

The red arrow indicates the optimal number of components in each plot

Supplementary Figure S4

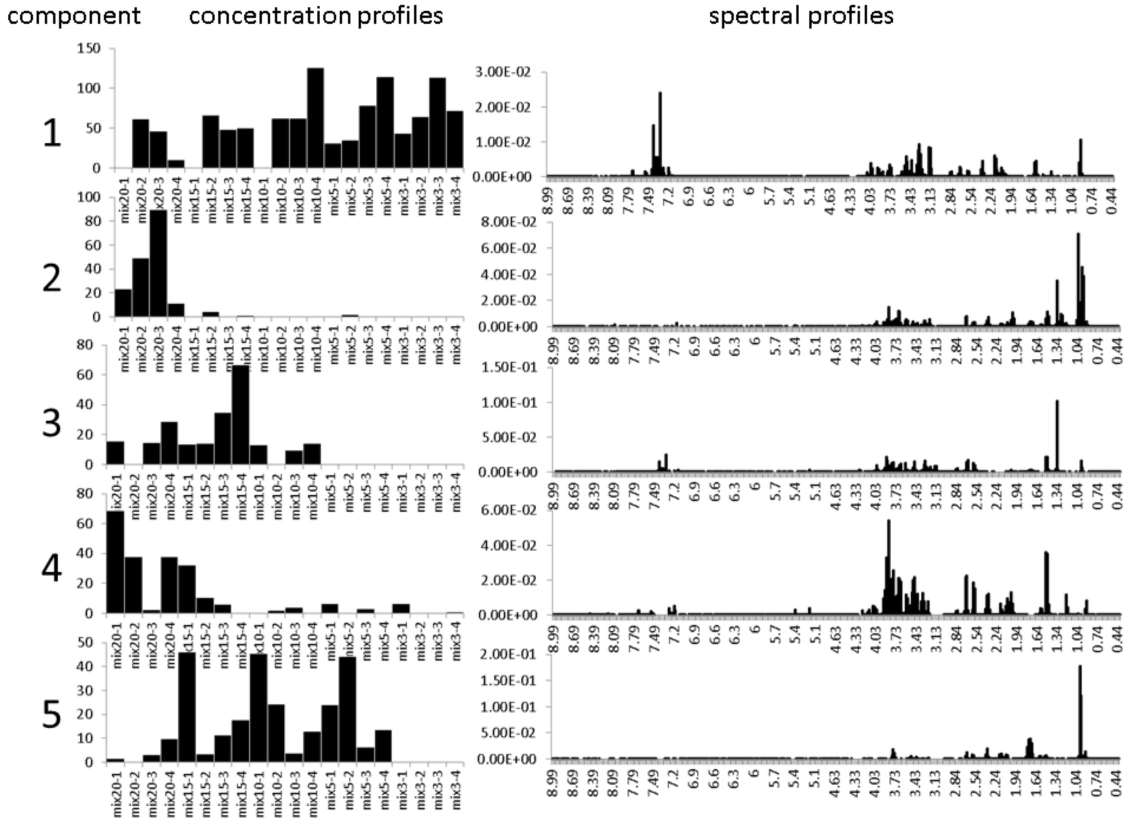

Results of conventional multivariate curve resolution-alternating least squares (MCR-ALS)

Concentration and spectral profiles of five components are shown. Scales of these graphs are in arbitrary units. Numbers in spectral profiles are chemical shift values of signals.

Supplementary Table S3

Reliable clusters and their elements (compound mixture analysis)

	Cluster ID	Minimum correlation coefficient	Size	Elements (Components)
1	cluster1_1	0.69	18	3C1,4C1,5C1,6C1,7C1,8C1,9C1,10C1,11C1,12C1,13C1,14C1,15C1,16C1,17C1,18C1,19C1,20C1
2	cluster1_2_2_2	0.92	7	12C5,13C5,14C5,15C5,16C5,17C5,20C5
3	cluster2_1_1_1	0.92	10	11C2,12C12,13C13,14C13,15C13,16C13,17C13,18C13,19C13,20C13
4	cluster2_1_2_1	0.67	8	11C10,13C10,14C10,15C10,16C16,17C16,18C16,19C16
5	cluster2_1_2_2	0.63	10	12C10,13C8,14C12,15C12,16C12,17C12,18C12,19C12,20C10,20C12
6	cluster2_2_1_1	0.95	7	14C6,15C6,16C6,17C6,18C6,19C6,20C6
7	cluster2_2_1_2_2	0.98	6	7C6,8C6,9C6,10C6,11C6,13C6
8	cluster2_2_1_2_4	0.88	6	14C2,15C15,16C15,17C15,19C19,20C18
9	cluster2_2_1_3_2	0.67	10	12C6,13C12,14C14,15C14,16C14,17C14,17C17,18C17,19C17,20C14
10	cluster2_2_2_1	0.73	16	5C3,6C3,7C7,8C8,9C7,10C7,11C7,12C7,13C7,14C7,15C7,16C7,17C7,18C7,19C7,20C20
11	cluster2_3_1_2	0.72	8	9C3,10C3,11C3,12C3,13C3,14C3,15C3,20C3
12	cluster2_3_2_1	0.95	17	4C4,5C2,6C2,7C2,8C2,9C2,10C2,11C11,12C11,13C11,14C11,15C11,16C11,17C11,18C11,19C11,20C11
13	cluster2_3_2_3	0.64	16	6C4,7C4,8C4,9C4,10C4,11C4,12C4,13C4,14C4,15C4,16C3,17C3,18C4,19C3,19C4,20C4
14	cluster2_3_2_4_1	0.87	8	4C3,6C5,7C3,8C7,9C9,10C9,11C9,12C9
15	cluster2_3_2_4_2	0.79	8	13C9,14C9,15C9,16C9,17C9,18C9,19C9,20C9

The characters in the column “Elements (components)” indicate the number of components and the component number. For example, 15C3 means component 3 when

multivariate curve resolution-alternating least squares (MCR-ALS) was calculated with the number of components set at 15.

Supplementary Figure S5

cluster1_1

1

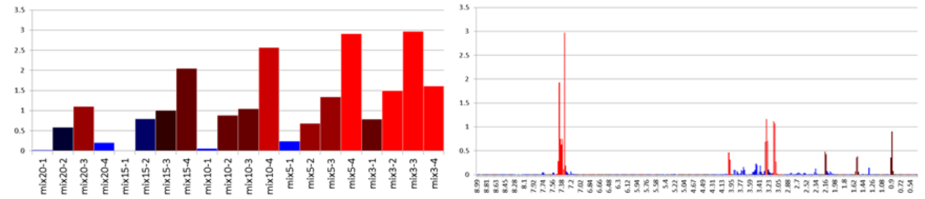

cluster1_2_2_2

2

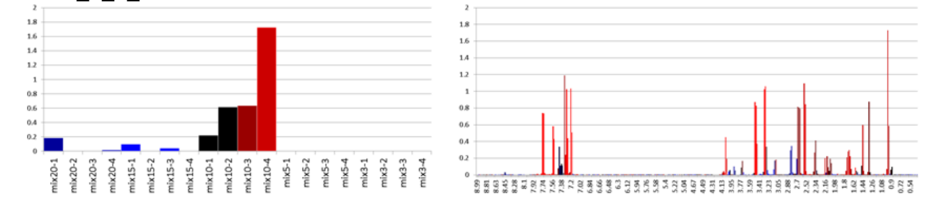

cluster2_1_1_1

3

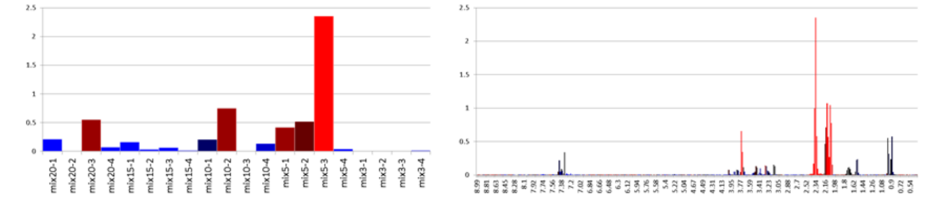

cluster2_1_2_1

4

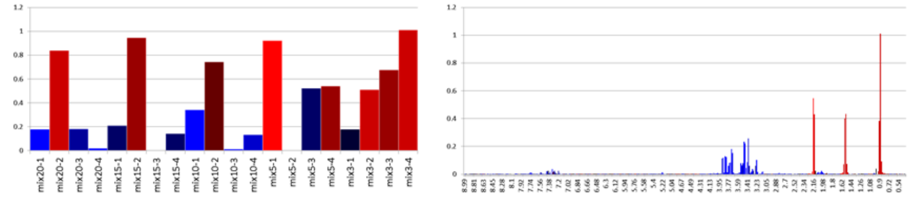

cluster2_1_2_2

5

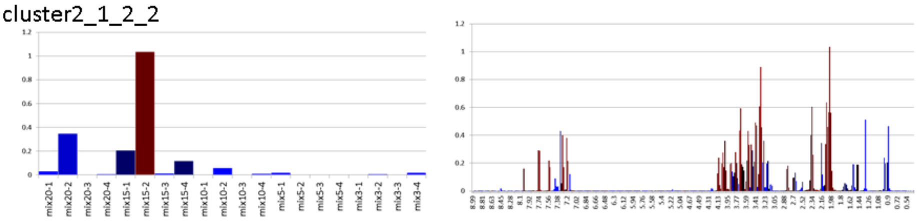

cluster2_2_1_1

6

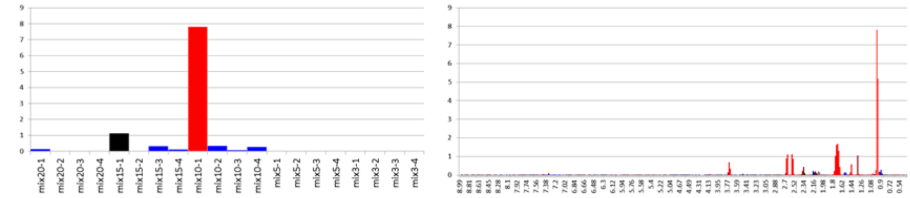

Supplementary Figure S5 (continued)

cluster2_2_1_2_2

7

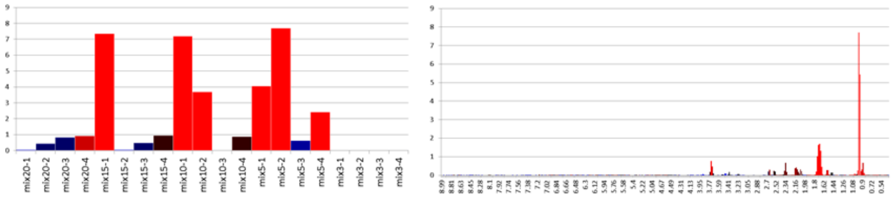

cluster2_2_1_2_4

8

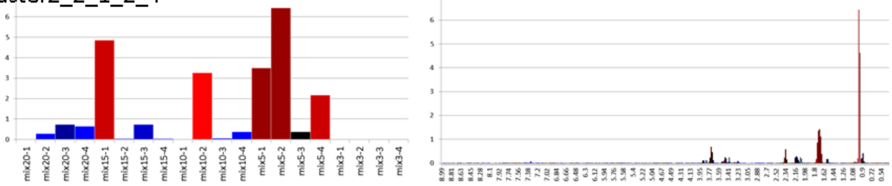

cluster2_2_1_3_2

9

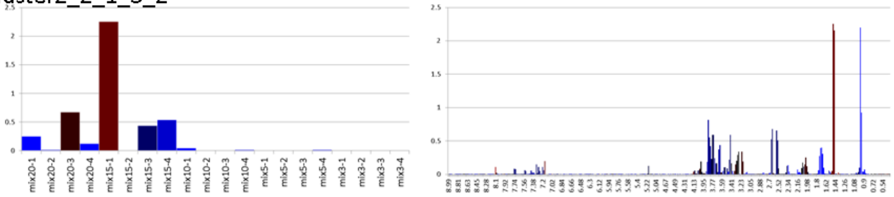

cluster2_2_2_1

10

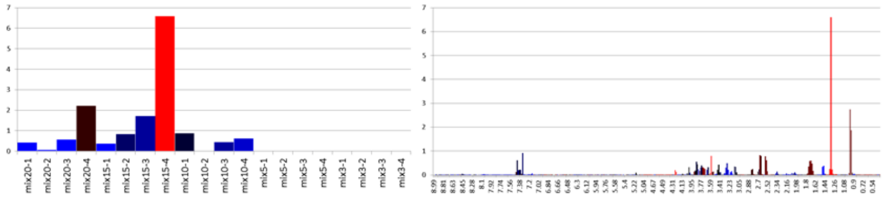

cluster2_3_1_2

11

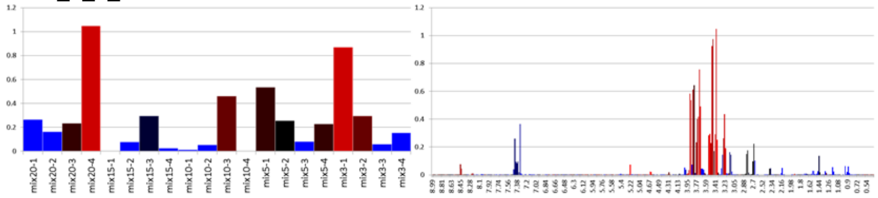

cluster2_3_2_1

12

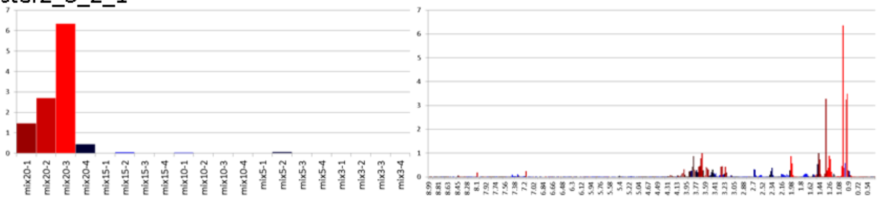

Supplementary Figure S5 (continued)

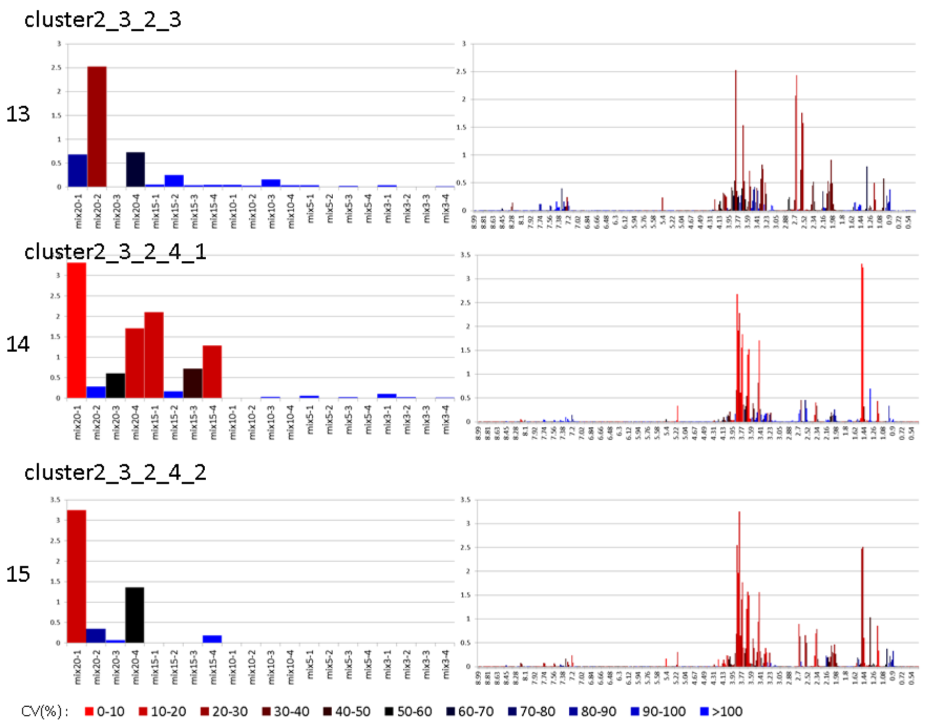

Supplementary Figure S5. The results of cluster aided multivariate curve resolution-alternating least squares (MCR-ALS).

Typical concentration and spectral profiles of 15 clusters are shown. Scales of these graphs are in arbitrary units.

Supplementary Table S4

Comparison of results of conventional and cluster-aided multivariate curve resolution-alternating least squares (MCR-ALS)

compound		Conventional MCR-ALS		Cluster-aided MCR-ALS	
		Related component (correlation coefficient)	corresponding signals found in spectral profiles (ppm)	Related component (correlation coefficient)	corresponding signals found in spectral profiles (ppm)
1	Phenylalanine	component 1 (0.82)	3.13, 3.27, 3.99, 7.32, 7.42	cluster1_1 (0.96)	3.13, 3.27, 3.99, 7.32, 7.42
2	Glutamate			cluster2_1_1_1 (0.91)	2.06, 2.12, 2.34, 3.75
3	Leucine	component 5 (0.98)	0.98, 1.74	cluster2_2_1_2_2 (0.95)	0.96, 1.72, 3.73
4	Tryptophan			cluster1_2_2_2 (0.83)	3.29, 3.31, 3.49, 4.05, 7.20, 7.28, 7.54, 7.74
5	Aspartate				
6	Histidine				
7	Proline			cluster2_1_2_2 (0.81)	2.00, 2.06, 2.34, 3.31, 3.39, 4.11
8	Alanine			cluster2_3_2_4_1 (0.97)	1.48, 3.75, 3.77
9	Threonine	component 3 (0.87)	1.34	cluster2_2_2_1 (0.86)	1.32, 3.57, 4.25
10	Isoleucine	component 2 (0.99)	0.94, 1.48, 2.00	cluster2_3_2_1 (0.99)	0.92, 1.00, 1.26, 1.46, 1.98, 3.65
11	Butyrate			cluster2_1_2_1 (0.86)	0.88, 1.56, 2.14
12	Formate				

13	Citrate				
14	Lactate				
15	Ethanol	component 4 (0.90)	1.20, 3.67	cluster2_3_2_4_2 (0.90)	1.18, 3.65
16	Malate				
17	Glucose			cluster2_3_1_2 (0.88)	3.23, 3.39, 3.45, 3.47, 3.69, 3.71, 3.81, 3.83, 3.89, 4.65, 5.22
18	Trehalose			cluster2_3_2_4_1 (0.98)	3.43, 3.63, 3.65, 3.75, 3.81, 3.83, 3.85, 3.87, 5.18
				cluster2_3_2_4_2 (0.81)	3.43, 3.45, 3.63, 3.65, 3.75, 3.77, 3.81, 3.83, 3.85, 5.18
19	Fructose				
20	Sucrose	component 4 (0.87)	3.45, 3.57, 3.65, 3.77, 3.83, 3.87, 3.91, 4.07, 5.40	cluster2_3_2_3 (0.83)	3.55, 3.67, 3.81, 3.89, 4.03, 4.05, 4.21, 5.40

Correlation coefficients were calculated between concentration of the compound and concentration profile of the result of MCR-ALS. Components with correlation coefficient of >0.8 are listed.

Supplementary Figure S6

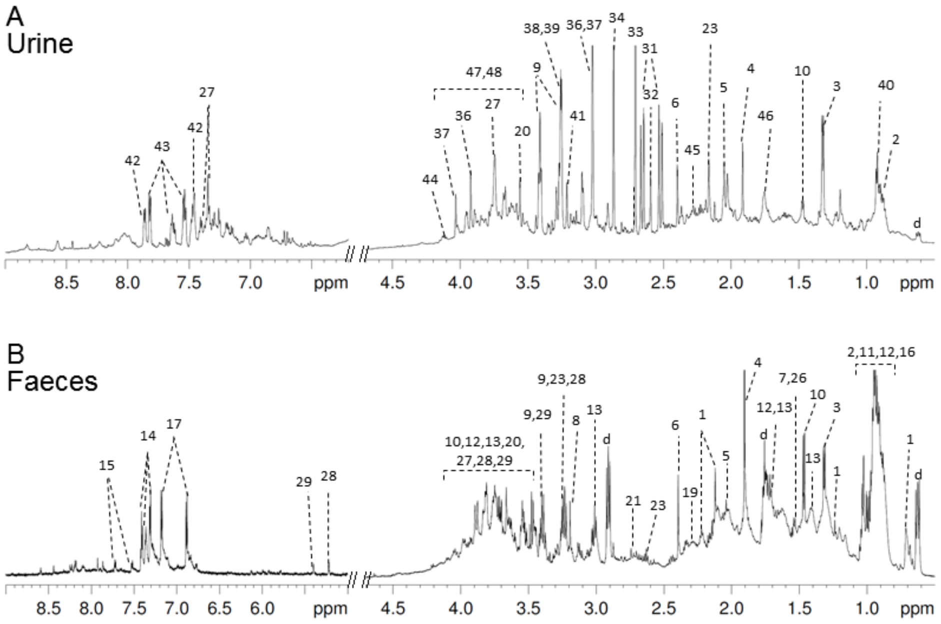

Supplementary Figure S6 Typical proton nuclear magnetic resonance (^{1}H-NMR) spectra of urine (A) and fecal (B) samples obtained from C57BL/6J control mice. Peak numbers refer to the metabolites listed in Supplementary Table S2.

Supplementary Table S5

List of metabolites found in urine and feces

Number	Metabolite	^{1}H chemical shift	^{13}C chemical shift	*P*-value (Urine)	*P*-value (Feces)	Urine(U) /Faeces(F)
1	Bile acid	0.71	14.8		3.1E-08	F
		0.91	24.8		3.0E-34	F
		1.32	35.1		1.9E-72	F
		1.41	38.1		2.6E-27	F
		1.68	49.4		4.2E-63	F
		1.72	35.0		1.3E-34	F
		1.92	30.0		9.6E-48	F
		2.10	37.2		2.3E-04	F
		2.23	37.2		5.3E-45	F
		0.97	19.5		2.2E-18	F
		4.06	76.1		5.6E-06	F
2	Butanoate	0.88	16.0	2.1E-43	9.2E-21	U,F
		2.15	42.3		1.6E-08	F
3	Lactate	1.31	22.7	5.4E-17	2.2E-47	U,F
		4.09	71.2	1.1E-48		U
4	Acetate	1.90	26.0	2.6E-11	7.3E-16	U,F
5	N-Acetyl group	2.04	24.7	2.8E-33	3.6E-01	U,F
6	Succinate	2.40	36.9	1.1E-28	7.3E-16	U,F
7	6-Carboxyhexanoate	1.29	31.4		1.9E-50	F
		1.56	28.4		3.7E-44	F
		2.18	40.2		1.1E-50	F
8	AcCt(N-CH_3)	3.18	56.5	1.5E-12	2.1E-02	U,F
9	Taurine	3.25	50.3	1.7E-22	6.1E-21	U,F
		3.41	38.3	5.1E-05	2.6E-21	U,F
10	L-Alanine	1.47	18.9	2.3E-79	2.5E-11	U,F
		3.77	53.2	4.8E-53	2.3E-79	U,F
11	L-Isoleucine	1.00	17.4		3.8E-08	F
		1.00	17.4		3.8E-08	F
		1.97	38.6		2.6E-02	F
12	L-Leucine	0.94	23.6		2.7E-11	F

		0.95	24.8		1.9E-12	F
		1.70	26.9		2.9E-10	F
		1.70	42.5		7.0E-07	F
		3.73	56.1		7.3E-04	F
13	L-Lysine	1.45	24.1		5.0E-10	F
		1.48	24.2		1.2E-07	F
		1.72	29.1		1.8E-08	F
		1.90	32.6		2.7E-10	F
		3.02	41.7		6.2E-17	F
		3.75	57.2		4.9E-06	F
14	L-Phenylalanine	3.98	58.8		7.5E-08	F
		7.32	132.0		4.5E-25	F
15	L-Tryptophan	7.53	114.7		6.1E-03	F
		7.72	121.2		5.7E-02	F
16	L-Valine	0.98	19.4		3.4E-09	F
		1.03	20.7		2.0E-12	F
		2.26	31.8		6.7E-10	F
17	L-Tyrosine	3.93	58.8		8.6E-13	F
		6.89	118.5		3.7E-07	F
18	L-Glutamine	2.13	29.0		7.6E-01	F
19	L-Glutamate	2.34	36.2		1.3E-04	F
20	L-Glycine	3.54	44.2	2.3E-35	7.6E-16	U,F
21	L-Aspartate	2.67	39.3		1.5E-06	F
		2.79	39.3		1.8E-04	F
		3.88	54.9		5.6E-20	F
22	L-Arginine	3.23	43.2		6.0E-03	F
23	L-Methionine	2.13	16.6	6.5E-135	1.1E-12	U,F
		2.63	31.5		2.2E-13	F
		3.85	56.6		9.6E-02	F
24	L-Asparagine	4.00	54.0		1.4E-04	F
25	L-Serine	3.83	59.1		9.5E-08	F
26	Alanyl-Alanine	1.35	19.8		6.7E-01	F
		1.54	19.2		8.2E-01	F
		4.14	54.0		1.8E-08	F
27		3.74	46.2	3.5E-11	4.7E-23	U,F

	Phenylacetylglycine	7.41	131.6	2.4E-02	1.6E-02	U,F
28	Glucose	3.24	76.8		2.8E-04	F
		3.40	72.3		3.1E-08	F
		3.45	78.7		5.5E-05	F
		3.53	74.2		2.1E-03	F
		3.71	75.5		1.3E-08	F
		3.72	63.4		7.1E-05	F
		3.76	63.3		1.7E-01	F
		3.82	74.2		3.2E-04	F
		3.83	63.3		4.7E-08	F
		3.89	63.4		2.3E-06	F
		4.64	98.6		7.2E-05	F
		5.23	94.8		1.8E-11	F
29	Sucrose	3.46	71.9		5.4E-07	F
		3.54	73.8		4.0E-09	F
		3.67	64.0		1.3E-09	F
		3.80	62.8		1.2E-06	F
		3.81	65.0		2.9E-01	F
		3.81	65.0		2.9E-01	F
		3.88	84.1		4.7E-01	F
		4.04	76.7		1.8E-07	F
		4.21	79.1		7.8E-04	F
30	2-oxoglutarate	2.43	33.2	2.6E-06		U
		2.99	38.5	3.3E-23		U
31	Citrate	2.54	48.4	4.2E-46		U
		2.64	48.5	2.6E-11		U
32	Methylamine	2.58	27.2	5.3E-57		U
33	Dimethylamine	2.70	37.1	2.3E-53		U
34	Trimethylamine	2.87	47.3	5.4E-10		U
35	N,N-dimethylglycine	2.92	46.3	5.2E-01		U
36	Creatine	3.03	39.6	4.3E-04		U
		3.92	56.5	4.9E-02		U
37	Creatinine	3.03	32.9	6.4E-05		U

		4.05	59.1	1.9E-28		U
38	Trimethylamine-N-oxide	3.26	62.3	3.0E-22		U
39	Betaine	3.25	56.1	2.4E-01		U
		3.89	68.9	8.1E-01		U
40	Isovaleric acid	0.90	24.7	HMDB		U
		2.04	49.9	HMDB		U
41	Carnitine	3.21	56.8	6.6E-13		U
42	Benzoate	7.47	131.0	6.0E-06		U
		7.54	134.0	HMDB		U
		7.86	131.5	1.8E-01		U
43	Hippurate	3.95	46.5	7.6E-43		U
		7.54	131.5	1.2E-01		U
		7.62	134.9	HMDB		U
		7.82	129.9	1.2E-01		U
44	Glycolate	3.94	64.0	2.6E-04		U
45	Acetylglutamate	2.22	36.8	6.2E-14		U
		4.09	58.0	6.7E-05		U
46	Putrescine	1.75	26.7	1.6E-04		U
47	Glucuronate	3.27	76.8	1.3E-05		U
		3.50	78.4	3.2E-16		U
		3.50	74.7	4.1E-09		U
		3.72	78.9	6.9E-04		U
		4.07	74.6	3.1E-11		U
		4.63	98.6	9.8E-09		U
		5.23	94.9	2.4E-03		U
48	Glyceric acid	3.73	66.9	HMDB		U
		3.82	66.9	HMDB		U
		4.11	76.2	HMDB		U
49	Ureidopropionic acid	2.37	40.1	HMDB		U
		3.29	39.9	HMDB		U

P-value corresponds to proximity between the measured and reference peak of the metabolite, as described by Chikayama et al [1]."HMDB" indicates that the metabolite was identified by HMDB (http://www.hmdb.ca/) database search [2, 3, 4].

References

1. Chikayama, E. *et al.* Statistical indices for simultaneous large-scale metabolite detections for a single NMR spectrum. *Anal. Chem.* **82**, 1653-1658 (2010).

2. Wishart, D. S. *et al.* HMDB: the Human Metabolome Database. *Nucleic Acids Res.* **35(Database issue)**, D521-D526 (2007).

3. Wishart, D. S. *et al.* HMDB: a knowledgebase for the human metabolome. *Nucleic Acids Res.* **37(Database issue)**, D603-D610 (2009).

4. Wishart, D. S. *et al.* HMDB 3.0 — The Human Metabolome Database in 2013. *Nucleic Acids Res.* **41(D1)**, D801-D807 (2013).

Supplementary Figure S7

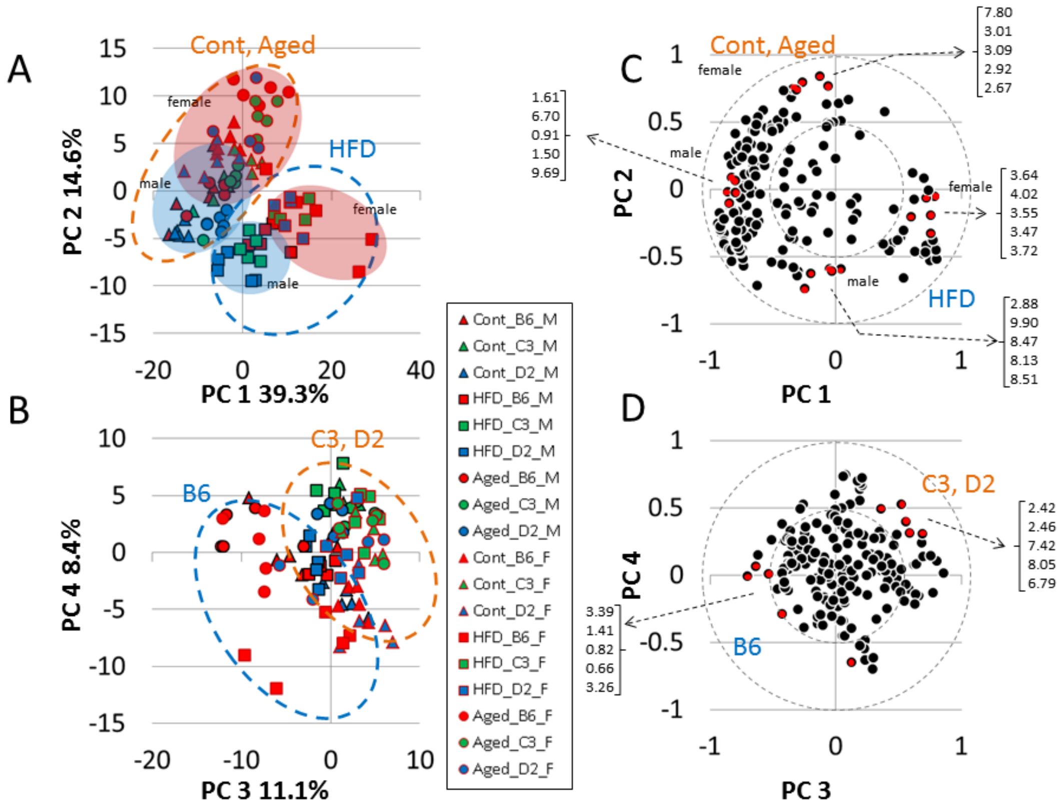

Supplementary Figure S7. The result of principal component analysis (urinary dataset). A. PC1-PC2 scores plot. A difference between high-fat-diet (HFD) feeding and normal-diet feeding was observed (indicated by a dotted line). Sex difference was also observed, which is indicated by blue (male) and red (female). B. PC3-PC4 scores plot. Strain difference between C57BL/6J (B6) and other strains are indicated by a dotted line. C. PC1-PC2 loadings plot. D. PC3-PC4 loadings plot. The numbers in panels C and D are the chemical shift values of typical five signals (ppm), located in the red-colored spots.

Supplementary Figure S8

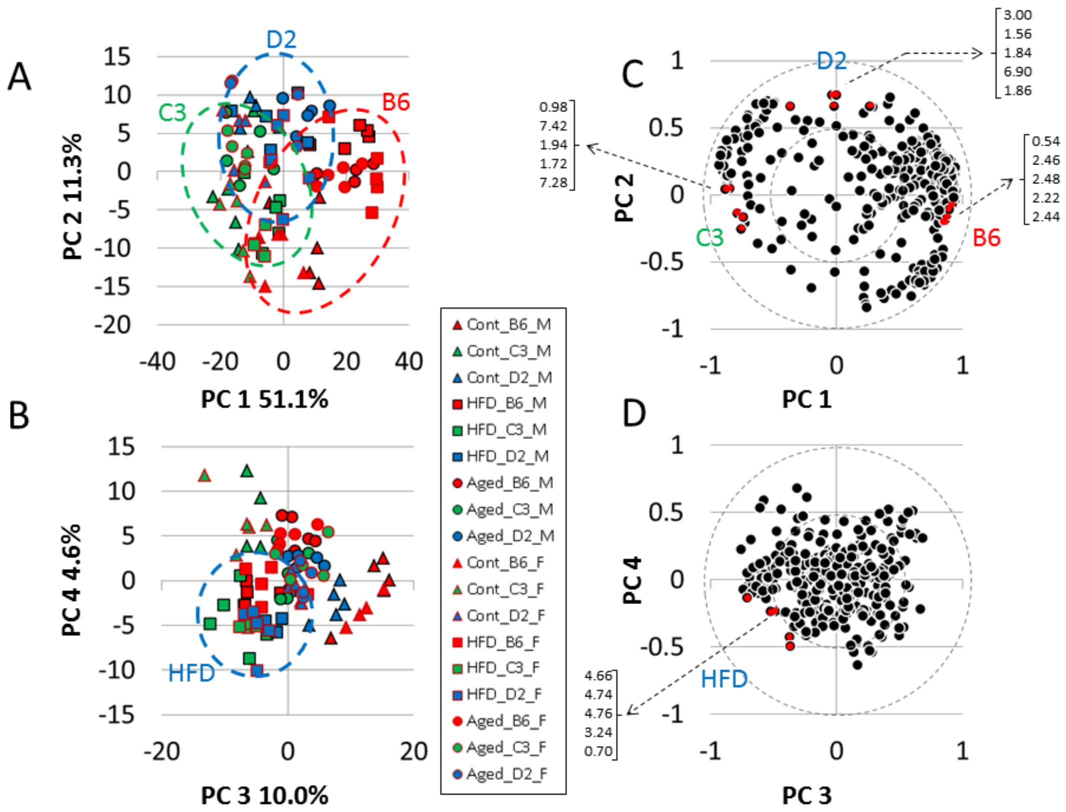

Supplementary Figure S8. The result of principal component analysis (fecal dataset). A. PC1-PC2 scores plot. B. PC3-PC4 scores plot. C. PC1-PC2 loadings plot. D. PC3-PC4 loadings plot. The numbers in panels C and D are the chemical shift values of typical five loading signals (ppm), located in the red-colored spots.

Supplementary Figure S9

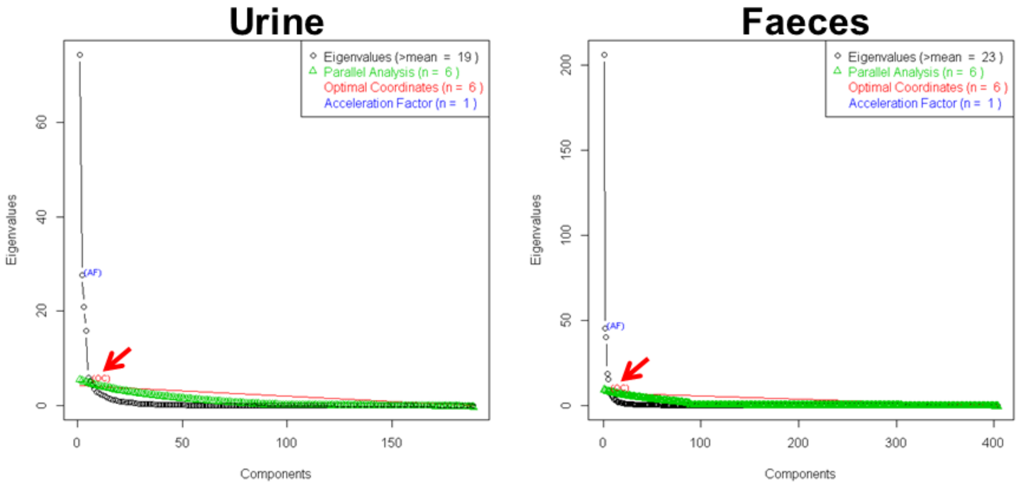

Supplementary Figure S9. Determination of the number of components for conventional multivariate curve resolution-alternating least squares (MCR-ALS) by parallel analysis.

The left and the right panels represent the scree plots of urine and feces, respectively. The red arrow indicates the optimal number of components in each plot.

Supplementary Figure S10

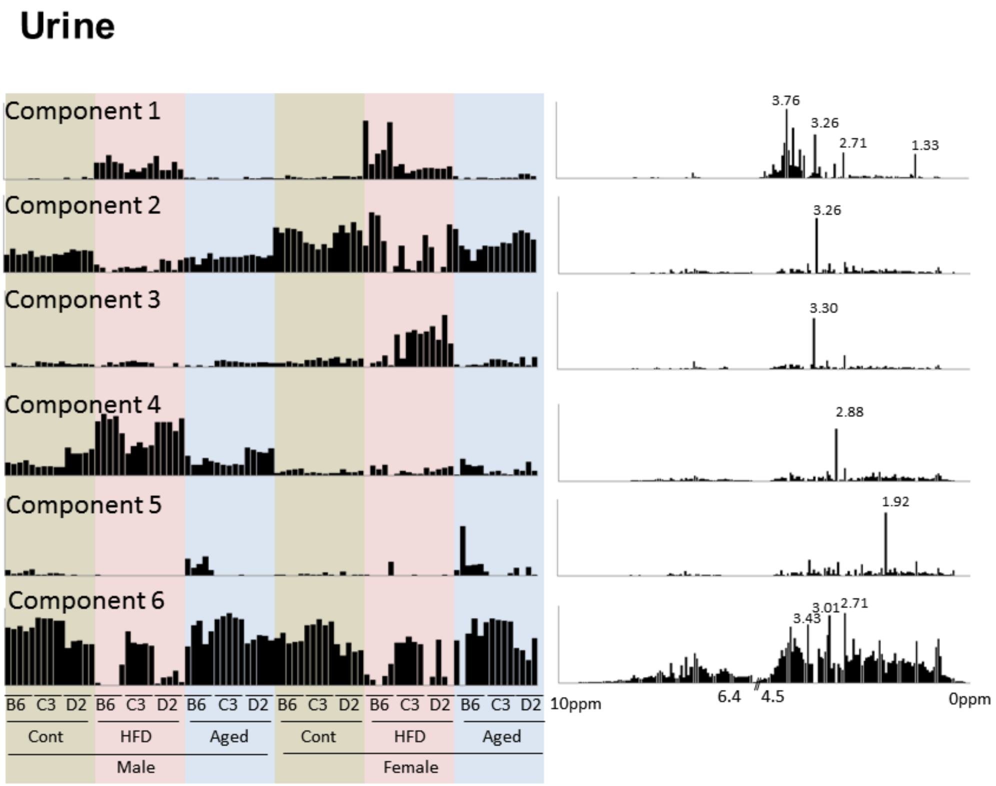

Supplementary Figure S10. Results of the conventional multivariate curve resolution-alternating least squares (MCR-ALS) (urinary dataset).

Concentration profiles and spectral profiles of six components are shown. Scales of these graphs are provided in arbitrary units. Numbers in spectral profiles are chemical shift values of signals.

Supplementary Figure S11

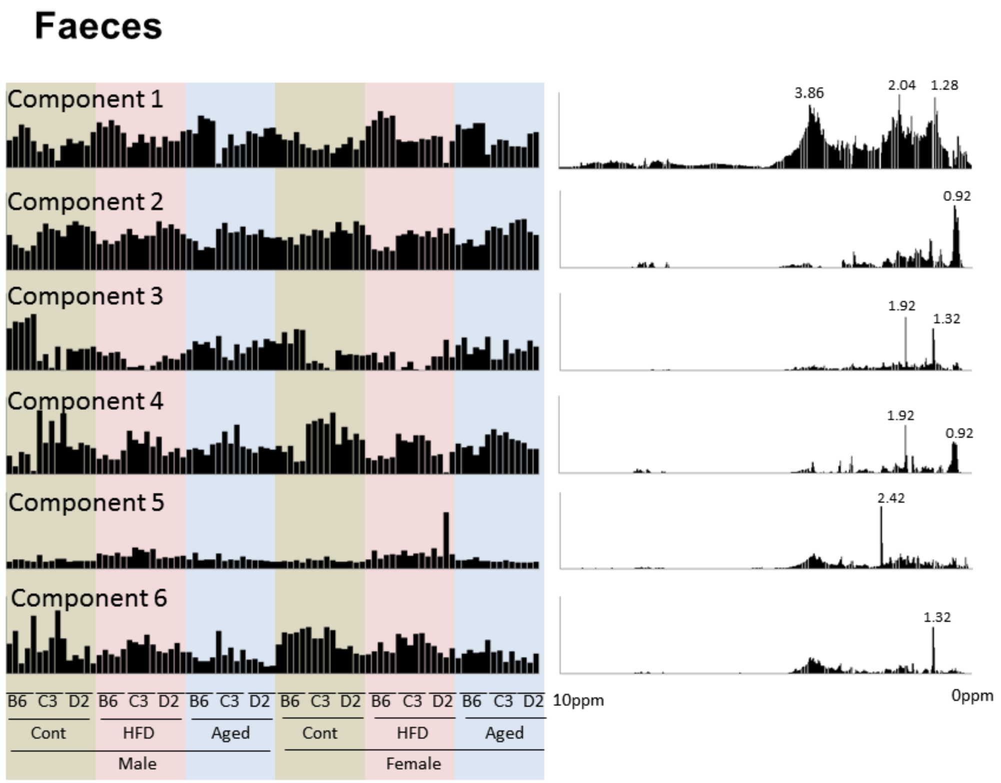

Supplementary Figure S11. Results of the conventional multivariate curve resolution-alternating least squares (MCR-ALS) (fecal dataset).

Concentration profiles and spectral profiles of six components are shown. Scales of these graphs are provided in arbitrary units. Numbers in spectral profiles are chemical shift values of signals.

Supplementary Table S6

Reliable clusters and their elements (urinary dataset analysis)

	Cluster ID	Minimum correlation coefficient	Size	Elements (Components)
1	Cluster67	0.63	89	2C1, 3C1, 4C4, 5C4, 6C4, 7C4, 8C4, 9C9, 10C9, 11C9, 12C9, 13C9, 14C9, 15C9, 16C9, 17C9, 18C9, 19C9, 20C9, 21C9, 22C9, 23C9, 24C9, 25C9, 26C9, 27C9, 28C9, 29C9, 30C9, 31C9, 31C16, 32C9, 33C9, 34C9, 35C9, 36C9, 37C9, 38C9, 39C9, 40C9, 41C9, 42C9, 43C9, 44C9, 45C9, 46C9, 47C9, 48C9, 49C9, 50C9, 51C9, 52C9, 53C9, 54C9, 55C9, 56C9, 57C9, 58C9, 59C9, 60C9, 61C9, 62C9, 63C9, 64C9, 65C9, 66C9, 67C9, 68C9, 69C9, 70C9, 71C9, 72C9, 73C9, 74C9, 75C9, 76C9, 77C9, 78C9, 79C9, 80C9, 81C9, 82C9, 83C9, 84C9, 85C9, 86C9, 87C9, 88C9, 89C9
2	Cluster124_1_2	0.60	86	9C7, 10C7, 11C8, 12C12, 13C12, 14C12, 15C12, 16C12, 17C12, 18C12, 19C12, 20C12, 21C12, 22C12, 23C12, 24C12, 25C12, 26C12, 27C12, 28C12, 29C12, 30C12, 31C12, 32C12, 33C12, 34C12, 35C12, 36C12, 37C12, 38C12, 39C12, 40C12, 41C12, 42C12, 43C12, 44C12, 45C12, 46C12, 47C12, 48C12, 49C12, 50C12, 51C12, 52C12, 53C12, 54C12, 55C12, 56C12, 57C12, 58C12, 59C12, 59C37, 60C12,

				60C37, 61C12, 61C37, 62C37, 63C12, 64C12, 65C12, 66C12, 67C12, 68C12, 69C37, 70C37, 71C12, 72C12, 73C12, 74C12, 75C12, 76C12, 77C12, 78C12, 79C12, 80C12, 81C12, 82C12, 83C12, 84C12, 85C12, 86C12, 86C75, 87C7, 87C75, 88C7, 89C7
3	Cluster136_2_1	0.77	84	6C5, 7C5, 8C5, 9C5, 10C5, 11C5, 12C5, 13C5, 14C5, 15C5, 16C5, 17C5, 18C5, 19C5, 20C5, 21C5, 22C5, 23C5, 24C5, 25C5, 26C5, 27C5, 28C5, 29C5, 30C5, 31C5, 32C5, 33C5, 34C5, 35C5, 36C5, 37C5, 38C5, 39C5, 40C5, 41C5, 42C5, 43C5, 44C5, 45C5, 46C5, 47C5, 48C5, 49C5, 50C5, 51C5, 52C5, 53C5, 54C5, 55C5, 56C5, 57C5, 58C5, 59C5, 60C5, 61C5, 62C5, 63C5, 64C5, 65C5, 66C5, 67C5, 68C5, 69C5, 70C5, 71C5, 72C5, 73C5, 74C5, 75C5, 76C5, 77C5, 78C5, 79C5, 80C5, 81C5, 82C5, 83C5, 84C5, 85C5, 86C5, 87C5, 88C5, 89C5
4	Cluster92_1_2	0.73	84	3C3, 4C3, 5C3, 6C3, 7C7, 10C8, 11C7, 12C7, 13C7, 14C7, 15C7, 16C7, 17C17, 18C17, 19C17, 20C17, 21C17, 57C26, 58C26, 59C26, 60C26, 61C26, 62C26, 64C26, 65C26, 66C26, 67C26, 68C26, 69C26, 70C26, 71C26, 72C26, 73C26, 74C3, 75C3, 76C3, 77C3, 78C3, 79C3, 80C3, 81C3, 82C3, 83C3, 84C3, 85C3, 86C3, 87C3, 88C3, 89C322C17, 23C17, 24C22,

				25C25, 26C26, 27C26, 28C26, 29C26, 30C26, 31C26, 32C26, 33C26, 34C26, 35C26, 36C26, 37C26, 38C26, 39C26, 40C26, 41C26, 42C26, 43C26, 44C26, 45C26, 46C26, 47C26, 48C26, 49C26, 50C26, 51C26, 52C26, 53C26, 54C26, 55C26, 56C26,
5	Cluster146_4_2_4_2_3_4_4	0.65	83	11C6, 12C6, 13C6, 14C6, 15C6, 16C6, 17C6, 18C18, 19C18, 20C18, 21C18, 22C18, 23C18, 24C18, 25C18, 26C18, 27C18, 28C18, 29C18, 30C18, 31C18, 32C32, 33C18, 34C18, 35C18, 36C32, 37C32, 38C32, 39C32, 40C32, 41C32, 42C32, 43C18, 44C18, 45C18, 46C18, 47C18, 48C18, 49C18, 50C18, 51C18, 52C32, 53C32, 54C32, 55C32, 56C32, 57C32, 58C32, 59C32, 60C32, 61C32, 62C32, 63C32, 64C32, 65C32, 66C32, 67C32, 68C32, 69C32, 70C32, 71C32, 72C32, 73C32, 74C32, 75C32, 76C24, 76C32, 77C24, 77C32, 78C24, 78C32, 79C24, 80C24, 81C24, 82C24, 83C18, 84C18, 84C24, 85C18, 86C18, 87C18, 88C18, 89C18
6	Cluster129_8	0.63	56	31C28, 32C28, 33C28, 34C28, 35C28, 36C28, 37C28, 38C28, 39C28, 40C28, 41C28, 42C28, 46C28, 47C28, 48C28, 49C28, 50C28, 51C28, 52C28, 53C28, 54C28, 55C28, 56C28, 57C28, 58C28, 59C28, 60C28, 61C28, 62C28, 63C28, 64C28, 65C28,

				66C28, 67C28, 68C28, 69C28, 70C28, 71C28, 72C28, 73C28, 74C28, 75C28, 76C28, 77C28, 78C28, 79C28, 80C28, 81C28, 82C28, 83C28, 84C28, 85C28, 86C28, 87C28, 88C28, 89C28
7	Cluster_62	0.79	50	35C14, 36C14, 37C14, 38C14, 39C14, 40C14, 41C14, 42C14, 43C14, 44C14, 45C14, 46C14, 47C14, 48C14, 49C14, 50C14, 51C14, 52C14, 53C14, 54C14, 55C14, 56C14, 57C14, 58C14, 59C14, 60C14, 61C14, 62C14, 63C14, 64C14, 65C14, 66C14, 67C14, 68C14, 69C14, 70C14, 74C14, 75C14, 78C14, 79C14, 80C14, 81C14, 82C14, 83C14, 84C14, 85C14, 86C14, 87C14, 88C14, 89C14
8	Cluster_71	0.74	41	43C23, 44C23, 45C23, 52C23, 53C23, 54C23, 55C23, 56C23, 57C23, 58C23, 59C23, 60C23, 61C23, 62C23, 63C23, 64C23, 65C23, 66C23, 67C23, 68C23, 69C23, 70C23, 71C23, 72C23, 73C23, 74C23, 75C23, 76C23, 77C23, 78C23, 79C23, 80C23, 81C23, 82C23, 83C23, 84C23, 85C23, 86C23, 87C23, 88C23, 89C23
9	Cluster137_2_4_5	0.79	38	28C24, 46C39, 47C39, 48C39, 49C39, 50C39, 51C39, 56C27, 57C27, 58C27, 59C27, 60C27, 61C27, 62C27, 63C27, 64C27, 65C27, 66C27, 67C27, 68C27, 69C27, 70C27, 71C27, 72C27, 76C27, 77C27, 78C27, 79C27,

				80C27, 81C27, 82C27, 83C27, 84C27, 85C27, 86C27, 87C27, 88C27, 89C27
10	Cluster150_5_8	0.81	34	43C25, 44C25, 45C25, 56C52, 57C52, 58C52, 59C52, 60C52, 61C52, 62C52, 63C52, 64C52, 65C52, 66C52, 67C52, 68C52, 69C52, 70C52, 71C52, 72C52, 73C52, 74C52, 75C52, 76C52, 77C52, 78C52, 79C52, 81C66, 84C52, 85C52, 86C52, 87C52, 88C52, 89C52
11	Cluster_30	0.93	34	29C1, 30C1, 31C1, 32C1, 33C1, 34C1, 35C1, 36C1, 37C1, 38C1, 39C1, 40C1, 41C1, 42C1, 43C1, 44C1, 45C1, 46C1, 47C1, 48C1, 49C1, 50C1, 51C1, 52C1, 53C1, 54C1, 55C1, 56C1, 57C1, 58C1, 59C1, 60C1, 61C1, 62C1
12	Cluster117_4	0.66	32	43C31, 44C31, 45C31, 46C31, 47C31, 48C31, 49C31, 50C31, 51C31, 52C31, 53C31, 54C31, 55C31, 59C31, 60C31, 61C31, 62C31, 63C31, 64C31, 65C31, 66C31, 67C31, 68C31, 69C31, 70C31, 71C31, 72C31, 73C31, 74C31, 75C31, 76C31, 77C31
13	Cluster136_2_2_2	0.70	27	63C56, 64C56, 65C56, 66C56, 67C56, 68C56, 69C56, 70C56, 71C56, 72C56, 73C56, 74C56, 75C56, 76C56, 77C56, 78C56, 79C56, 80C56, 81C56, 82C56, 83C56, 84C56, 85C56, 86C56, 87C56, 88C56, 89C56
14	Cluster85	0.66	27	63C63, 64C63, 65C63, 66C63, 67C63, 68C63, 69C63, 70C63,

				71C63, 72C63, 73C63, 74C63, 75C63, 76C63, 77C63, 78C63, 79C63, 80C63, 81C63, 82C63, 83C63, 84C63, 85C63, 86C63, 87C63, 88C63, 89C63
15	Cluster150_5_6	0.77	26	23C22, 24C21, 25C21, 26C21, 27C27, 28C23, 29C23, 30C23, 31C23, 32C23, 33C23, 34C23, 35C23, 36C23, 37C23, 38C23, 39C23, 40C23, 41C23, 42C23, 46C23, 47C23, 48C23, 49C23, 50C23, 51C23
16	Cluster_35	0.92	26	63C1, 64C1, 65C1, 66C1, 67C1, 68C1, 69C1, 70C1, 71C1, 72C1, 73C1, 74C1, 75C1, 76C1, 77C1, 78C1, 80C1, 81C1, 82C1, 83C1, 84C1, 85C1, 86C1, 87C1, 88C1, 89C1
17	Cluster_78	0.78	25	58C58, 59C58, 60C58, 61C58, 62C58, 63C58, 64C58, 65C58, 66C58, 67C58, 68C58, 69C58, 70C58, 78C72, 79C72, 80C72, 81C72, 82C72, 83C72, 84C72, 85C72, 86C72, 87C72, 88C72, 89C72
18	Cluster72_6	0.76	24	28C21, 29C21, 30C21, 31C21, 32C21, 33C21, 34C21, 35C21, 36C21, 37C21, 38C21, 39C21, 40C21, 41C21, 42C21, 43C21, 44C21, 45C21, 46C21, 47C21, 48C21, 49C21, 50C21, 51C21
19	Cluster144_5	0.66	23	36C34, 37C34, 38C34, 39C34, 40C34, 41C34, 42C34, 43C34, 44C34, 45C34, 46C34, 47C34, 48C34, 49C34, 50C34, 51C34, 52C34, 53C34, 54C34, 55C34, 56C34, 57C34, 58C34

20	Cluster107_7	0.69	23	24C17, 35C33, 36C35, 37C35, 38C35, 39C35, 40C35, 41C41, 42C41, 43C41, 44C41, 45C41, 46C41, 47C41, 48C41, 49C41, 50C41, 51C41, 52C41, 53C53, 54C41, 55C41, 70C57
21	Cluster76	0.66	23	33C22, 34C22, 36C22, 37C22, 38C22, 39C22, 40C22, 41C22, 42C22, 43C22, 44C22, 45C22, 46C22, 47C22, 48C22, 49C22, 50C22, 51C22, 63C26, 75C53, 80C53, 81C53, 82C53

The characters in the column of "Elements (Components)" indicate the number of components and the component number. For example, 15C3 means component 3 when multivariate curve resolution-alternating least squares (MCR-ALS) was calculated with the number of components set at 15.

Supplementary Table S7

Reliable clusters and their elements (fecal dataset analysis)

	Cluster ID	Minimum correlation coefficient	Size	Elements of cluster (Components)
1	Cluster176	0.76	89	1C1, 2C2, 3C2, 4C2, 5C2, 6C2, 7C2, 8C2, 9C2, 10C2, 11C2, 12C2, 13C2, 14C2, 15C2, 16C2, 17C2, 18C2, 19C2, 20C2, 21C2, 22C2, 23C2, 24C2, 25C2, 26C2, 27C2, 28C2, 29C2, 30C2, 31C2, 32C2, 33C2, 34C2, 35C2, 36C2, 37C2, 38C2, 39C2, 40C2, 41C2, 42C2, 43C2, 44C2, 45C2, 46C2, 47C2, 48C2, 49C2, 50C2, 51C2, 52C2, 53C2, 54C2, 55C2, 56C2, 57C2, 58C2, 59C2, 60C2, 61C2, 62C2, 63C2, 64C2, 65C2, 66C2, 67C2, 68C2, 69C2, 70C2, 71C2, 72C2, 73C2, 74C2, 75C2, 76C2, 77C2, 78C2, 79C2, 80C2, 81C2, 82C2, 83C2, 84C2, 85C2, 86C2, 87C2, 88C2, 89C2
2	Cluster220_2_1	0.63	86	6C5, 7C7, 8C7, 9C9, 10C10, 11C10, 12C12, 13C12, 14C12, 15C12, 16C10, 17C10, 18C10, 19C10, 20C10, 21C10, 22C10, 23C10, 24C10, 25C10, 26C10, 27C10, 28C10, 29C10, 30C10, 31C10, 32C10, 33C10, 34C10, 35C10, 36C36, 37C36, 38C36, 39C22, 40C22, 41C22, 42C22, 43C22, 44C22, 45C22, 46C22, 47C36, 48C36, 49C22, 50C22, 51C22, 52C22, 53C36, 54C36, 55C36, 56C36, 57C10, 58C10, 59C10,

				60C10, 61C61, 62C61, 63C61, 64C61, 65C61, 66C61, 67C61, 68C10, 68C63, 69C63, 70C63, 71C63, 72C63, 73C63, 74C63, 75C63, 76C63, 77C63, 78C63, 79C10, 80C10, 81C10, 82C10, 83C10, 84C10, 85C10, 86C10, 87C10, 88C10, 89C9, 89C17
3	Cluster189	0.64	86	5C4, 6C4, 7C4, 8C4, 9C4, 10C4, 11C4, 12C4, 13C4, 14C4, 15C4, 16C4, 17C4, 18C4, 19C4, 20C4, 21C4, 22C4, 23C4, 24C4, 25C4, 26C4, 27C4, 28C4, 29C4, 30C4, 31C4, 32C4, 33C4, 34C4, 35C4, 36C4, 37C4, 38C4, 39C4, 40C4, 41C4, 42C4, 43C4, 44C4, 45C4, 46C4, 47C4, 48C4, 49C4, 50C4, 51C4, 52C4, 53C4, 54C4, 55C4, 56C4, 57C4, 58C4, 59C4, 60C4, 61C4, 62C4, 63C4, 64C4, 65C4, 66C4, 67C4, 68C4, 69C4, 70C4, 71C4, 72C4, 73C4, 74C4, 75C4, 76C4, 77C4, 78C4, 79C4, 80C4, 81C4, 82C4, 83C4, 84C4, 85C4, 86C4, 87C4, 88C4, 89C4, 89C76
4	Cluster215_2	0.78	66	3C3, 5C3, 8C3, 9C3, 10C3, 11C3, 12C3, 30C16, 31C16, 32C16, 33C16, 34C16, 35C16, 36C16, 37C16, 38C16, 39C16, 40C16, 41C16, 42C16, 43C16, 44C16, 45C16, 46C16, 47C16, 48C16, 49C16, 50C16, 51C16, 52C16, 53C16, 54C16, 55C16, 56C16, 57C16, 58C16, 59C16, 60C16, 61C16, 62C16, 63C16, 64C16, 65C16, 66C16, 67C16, 68C16, 69C16,

				70C16, 71C16, 72C16, 73C16, 74C16, 75C16, 76C16, 77C16, 78C16, 79C16, 80C16, 81C16, 82C16, 83C16, 84C16, 85C16, 86C16, 87C16, 88C16
5	Cluster173	0.75	59	30C30, 31C30, 32C30, 33C30, 34C30, 35C30, 36C30, 37C30, 38C30, 39C30, 40C30, 41C30, 42C30, 43C30, 44C30, 45C30, 46C30, 47C30, 48C30, 49C30, 50C30, 51C30, 52C30, 53C30, 54C30, 55C30, 56C30, 57C30, 58C30, 59C30, 60C30, 61C30, 62C30, 63C30, 64C30, 65C30, 66C30, 67C30, 68C30, 69C30, 70C30, 71C30, 72C30, 73C30, 74C30, 75C30, 76C30, 77C30, 78C30, 79C30, 80C30, 81C30, 82C30, 83C30, 84C30, 85C30, 86C30, 87C30, 88C30
6	Cluster185	0.69	57	32C32, 33C32, 34C32, 35C35, 36C35, 37C35, 38C35, 39C35, 40C35, 41C35, 42C35, 43C35, 44C35, 45C35, 46C35, 47C35, 48C35, 49C35, 50C35, 51C35, 52C35, 53C35, 54C35, 55C35, 56C35, 57C35, 58C35, 59C35, 60C35, 61C35, 62C35, 63C35, 64C35, 65C35, 66C35, 67C35, 68C35, 69C35, 70C35, 71C35, 72C35, 73C35, 74C35, 75C35, 76C35, 77C35, 78C35, 79C35, 80C35, 81C35, 82C35, 83C35, 84C35, 85C35, 86C35, 87C35, 88C35

7	Cluster174	0.80	50	39C20, 40C20, 41C20, 42C20, 43C20, 44C20, 45C20, 46C20, 47C20, 48C20, 49C20, 50C20, 51C20, 52C20, 53C20, 54C20, 55C20, 56C20, 57C20, 58C20, 59C20, 60C20, 61C20, 62C20, 63C20, 64C20, 65C20, 66C20, 67C20, 68C20, 69C20, 70C20, 71C20, 72C20, 73C20, 74C20, 75C20, 76C20, 77C20, 78C20, 79C20, 80C20, 81C20, 82C20, 83C20, 84C20, 85C20, 86C20, 87C20, 88C20
8	Cluster202	0.65	41	49C49, 50C49, 51C49, 52C49, 53C49, 54C49, 55C49, 56C49, 57C49, 58C49, 59C49, 60C49, 61C49, 62C49, 63C49, 64C49, 65C49, 66C49, 67C49, 68C49, 69C49, 70C49, 71C49, 72C49, 73C49, 74C49, 75C49, 76C49, 77C49, 78C49, 79C49, 80C49, 81C49, 82C49, 83C49, 84C49, 85C49, 86C49, 87C49, 88C49, 89C85
9	Cluster180	0.76	36	53C8, 54C8, 55C8, 56C8, 57C8, 58C8, 59C8, 60C8, 61C8, 62C8, 63C8, 64C8, 65C8, 66C8, 67C8, 68C8, 69C8, 70C8, 71C8, 72C8, 73C8, 74C8, 75C8, 76C8, 77C8, 78C8, 79C8, 80C8, 81C8, 82C8, 83C8, 84C8, 85C8, 86C8, 87C8, 88C8
10	Cluster188	0.73	36	53C19, 54C19, 55C19, 56C19, 57C19, 58C19, 59C19, 60C19, 61C19, 62C19, 63C19, 64C19, 65C19, 66C19, 67C19, 68C19,

				69C19, 70C19, 71C19, 72C19, 73C19, 74C19, 75C19, 76C19, 77C19, 78C19, 79C19, 80C19, 81C19, 82C19, 83C19, 84C19, 85C19, 86C19, 87C19, 88C19
11	Cluster178	0.79	32	57C39, 58C39, 59C39, 60C39, 61C39, 62C39, 63C39, 64C39, 65C39, 66C39, 67C39, 68C39, 69C39, 70C39, 71C39, 72C39, 73C39, 74C39, 75C39, 76C39, 77C39, 78C39, 79C39, 80C39, 81C39, 82C39, 83C39, 84C39, 85C39, 86C39, 87C39, 88C39
12	Cluster199	0.63	32	42C42, 43C42, 44C42, 45C42, 46C42, 47C42, 48C42, 49C42, 50C42, 51C42, 52C42, 53C42, 54C42, 55C42, 56C42, 57C42, 58C42, 59C42, 60C42, 61C42, 62C42, 63C42, 64C42, 65C42, 66C42, 67C42, 68C42, 69C42, 70C42, 71C42, 72C42, 73C42
13	Cluster164	0.86	26	19C6, 20C6, 21C6, 22C6, 23C6, 24C6, 25C6, 26C6, 27C6, 28C6, 29C6, 30C6, 31C6, 32C6, 33C6, 34C6, 35C6, 36C6, 37C6, 38C6, 39C6, 40C6, 41C6, 42C6, 43C6, 44C6
14	Cluster210_2_6	0.87	25	45C40, 46C40, 47C25, 48C25, 49C25, 50C25, 51C25, 52C25, 53C25, 54C25, 55C25, 56C25, 57C25, 58C25, 59C25, 60C25, 68C25, 76C25, 77C25, 79C25, 80C25, 82C25, 84C25, 85C25, 86C25
15	Cluster224_10	0.73	22	63C25, 64C25, 65C25, 66C25, 68C33, 69C33, 70C33, 71C33,

				72C33, 73C33, 74C33, 75C33, 79C65, 80C65, 81C65, 82C65, 83C65, 84C65, 85C65, 86C65, 87C65, 89C32
16	Cluster192	0.61	22	67C67, 68C67, 69C67, 70C67, 71C67, 72C67, 73C67, 74C67, 75C67, 76C67, 77C67, 78C67, 79C67, 80C67, 81C67, 82C67, 83C67, 84C67, 85C67, 86C67, 87C67, 88C67
17	Cluster197	0.70	20	69C68, 70C68, 71C68, 72C68, 73C68, 74C68, 75C68, 76C68, 77C68, 78C68, 79C68, 80C68, 81C68, 82C68, 83C68, 84C68, 85C68, 86C68, 87C68, 88C68
18	Cluster200	0.69	19	53C32, 54C32, 55C32, 56C32, 57C32, 58C32, 59C32, 60C32, 61C32, 62C32, 63C32, 64C32, 65C32, 66C32, 67C32, 68C32, 69C32, 70C32, 71C32
19	Cluster228_10	0.85	18	69C9, 70C9, 71C9, 74C9, 75C9, 76C9, 77C9, 78C9, 79C9, 80C9, 81C9, 82C9, 83C9, 84C9, 85C9, 86C9, 87C9, 88C9
20	Cluster171	0.83	18	39C39, 40C39, 41C39, 42C39, 43C39, 44C39, 45C39, 46C39, 47C39, 48C39, 49C39, 50C39, 51C39, 52C39, 53C39, 54C39, 55C39, 56C39
21	Cluster215_3	0.76	17	13C13, 14C13, 15C13, 16C16, 17C16, 18C16, 19C16, 20C16, 21C16, 22C16, 23C16, 24C16, 25C16, 26C16, 27C16, 28C16, 29C16
22	Cluster210_2_7	0.75	17	28C22, 29C22, 36C10, 61C10, 62C10, 63C10, 64C10, 65C10,

				66C10, 69C10, 70C10, 71C10, 72C10, 73C10, 75C10, 83C73, 87C81
23	Cluster227_2_15	0.82	16	72C27, 73C27, 74C27, 75C27, 76C27, 77C27, 78C27, 79C27, 80C27, 81C27, 82C27, 83C27, 84C27, 85C27, 86C27, 87C27
24	Cluster137	0.91	15	53C53, 54C53, 55C53, 56C53, 57C53, 58C53, 59C53, 60C53, 61C53, 62C53, 63C53, 64C53, 65C53, 66C53, 67C53
25	Cluster203_9	0.69	14	3C1, 4C1, 5C1, 6C1, 7C1, 8C1, 9C1, 10C1, 11C1, 12C1, 13C1, 14C1, 15C1, 16C1
26	Cluster214_9	0.83	14	53C18, 54C18, 55C18, 56C18, 57C18, 58C18, 59C18, 60C18, 61C18, 62C18, 63C18, 64C18, 65C18, 66C18
27	Cluster155	0.90	14	53C14, 54C14, 55C14, 56C14, 57C14, 58C14, 59C14, 60C14, 61C14, 62C14, 63C14, 64C14, 65C14, 66C14
28	Cluster194	0.75	14	75C75, 76C75, 77C75, 78C75, 79C75, 80C75, 81C75, 82C75, 83C75, 84C75, 85C75, 86C75, 87C75, 88C75
29	Cluster195	0.68	14	17C3, 18C3, 19C3, 20C3, 21C3, 22C3, 23C3, 24C3, 25C3, 26C3, 27C3, 28C3, 29C3, 30C3
30	Cluster225_1	0.70	13	76C76, 77C76, 78C76, 79C76, 80C76, 81C76, 82C76, 83C76, 84C76, 85C76, 86C76, 87C76, 88C76
31	Cluster219_1_19	0.92	12	57C11, 58C11, 59C11, 60C11, 61C11, 62C11, 63C11, 64C11, 65C11, 66C11, 67C11, 68C11

32	Cluster207_3_6	0.94	12	57C15, 58C15, 59C15, 60C15, 61C15, 62C15, 63C15, 64C15, 65C15, 66C15, 67C15, 68C15
33	Cluster207_3_8	0.91	12	45C15, 46C15, 47C15, 48C15, 49C15, 50C15, 51C15, 52C15, 53C15, 54C15, 55C15, 56C15
34	Cluster231_6	0.64	12	76C40, 77C40, 78C40, 79C40, 80C40, 81C40, 82C40, 83C40, 84C40, 85C40, 86C40, 87C40
35	Cluster231_7	0.68	12	60C40, 61C40, 62C40, 63C40, 64C40, 65C40, 66C40, 67C40, 68C40, 69C40, 70C40, 71C40

The characters in the column of "Elements (Components)" indicate the number of components and the component number. For example, 15C3 means component 3 when multivariate curve resolution-alternating least squares (MCR-ALS) was calculated with the number of components set at 15.

Supplementary Figure S12

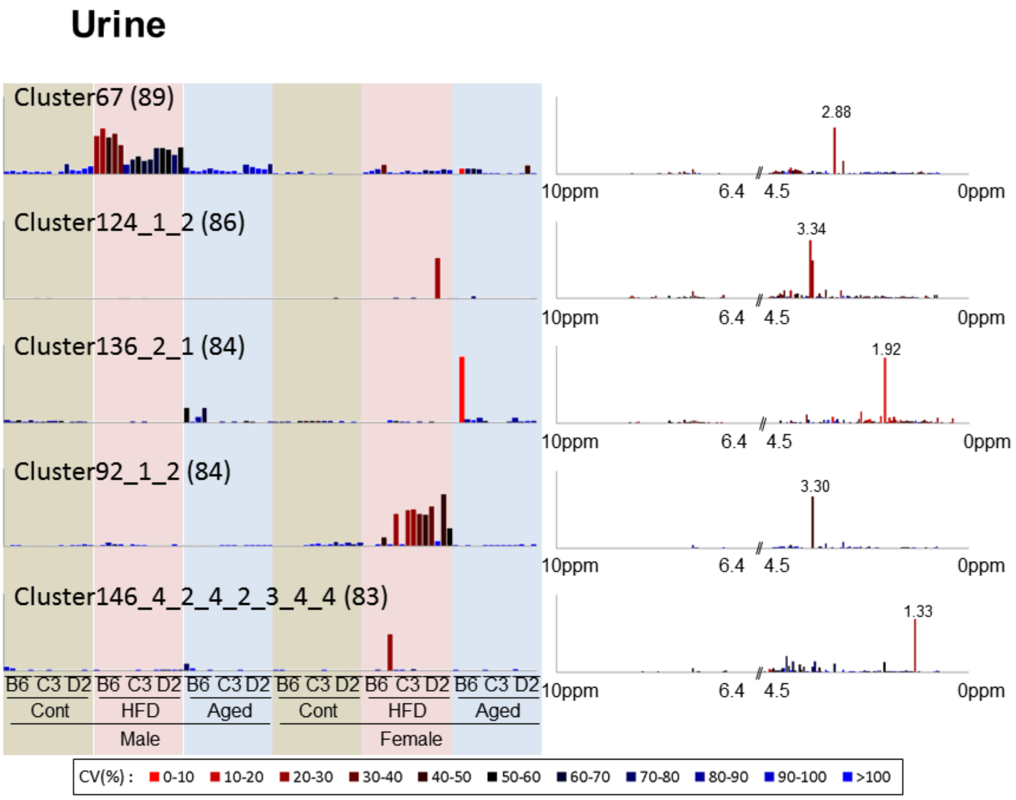

Supplementary Figure S12 (continued)

Urine

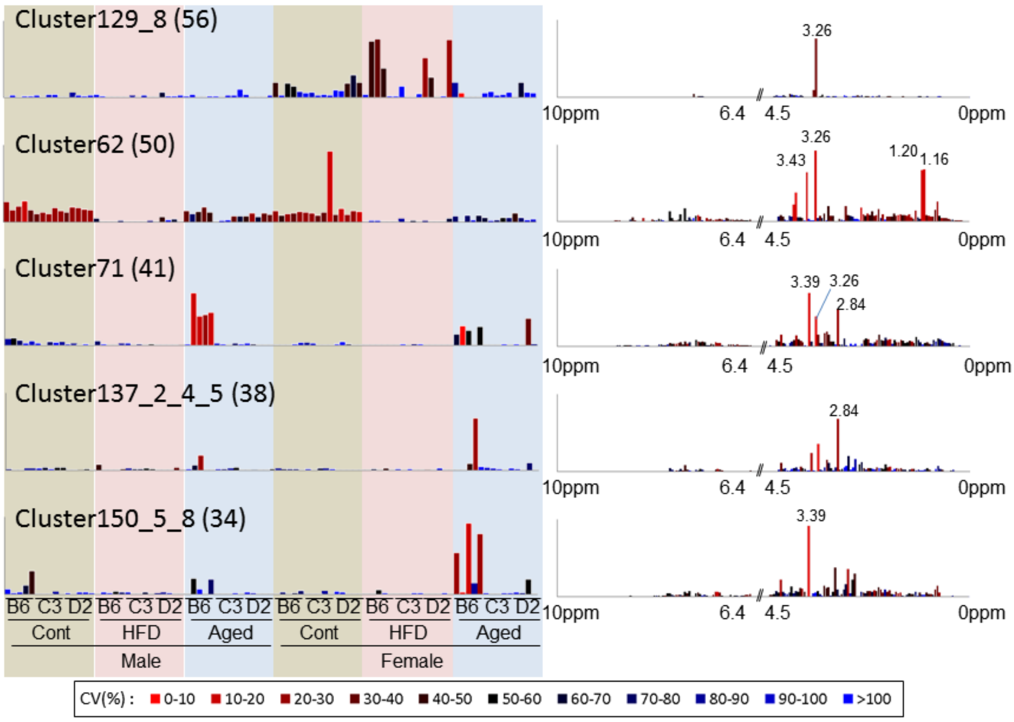

Supplementary Figure S12 (continued)

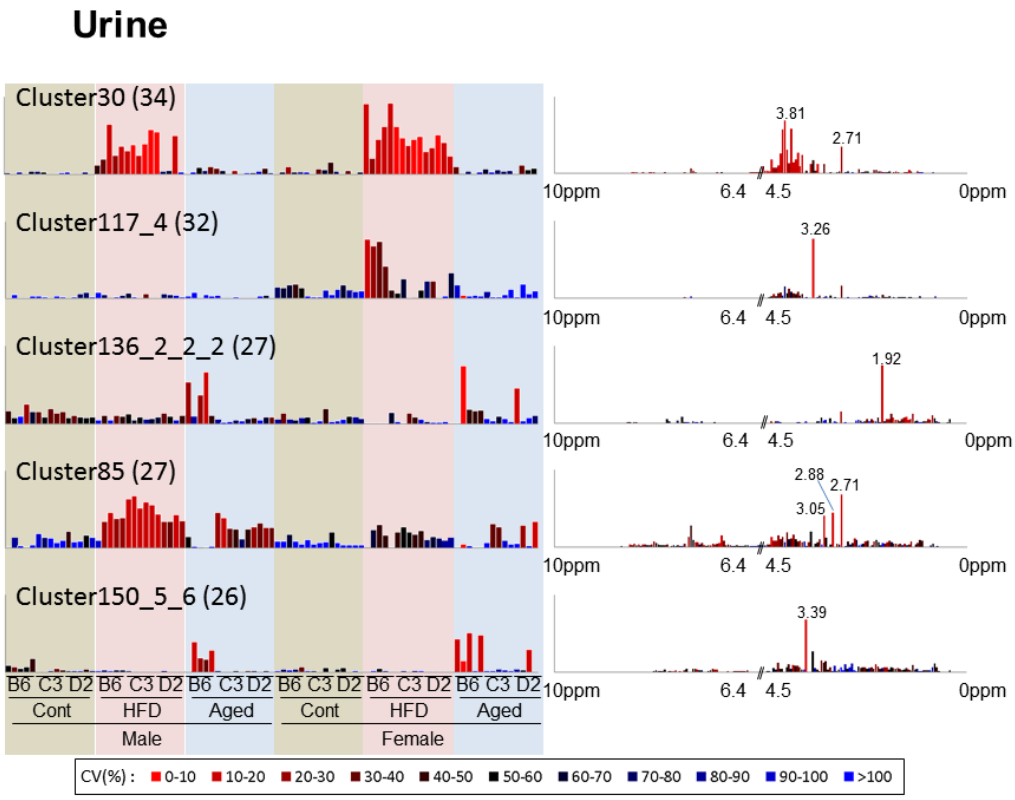

Supplementary Figure S12 (continued)

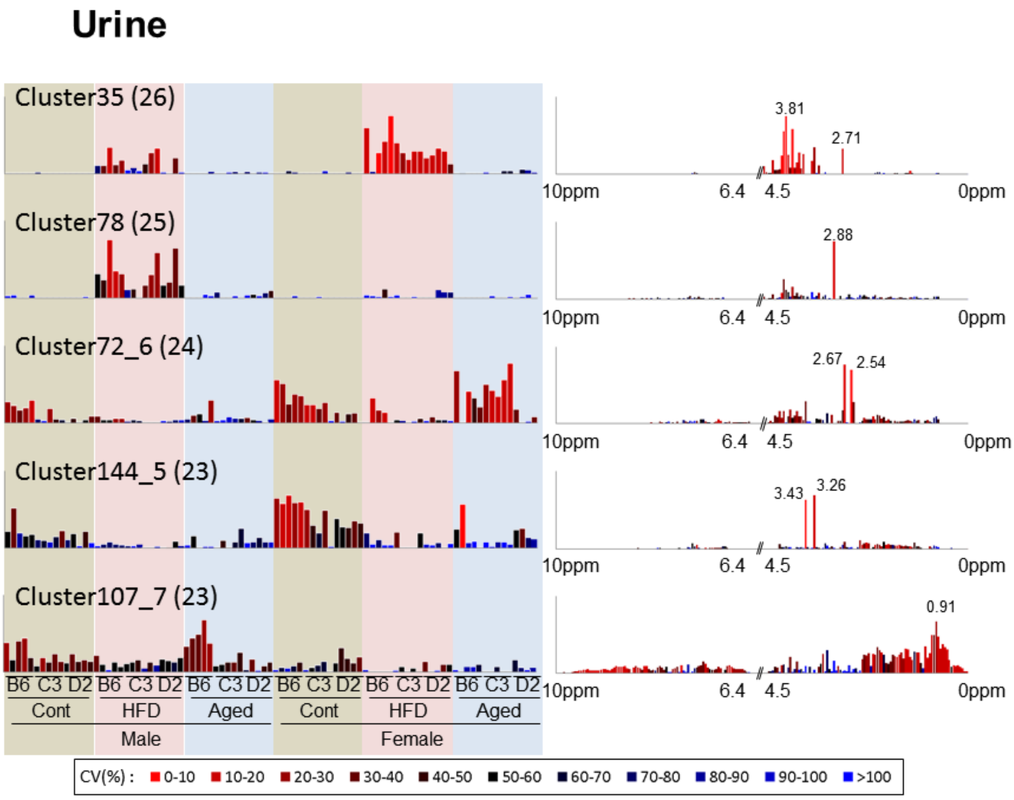

Supplementary Figure S12 (continued)

Urine

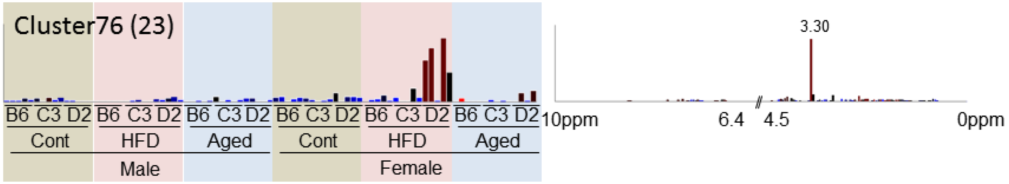

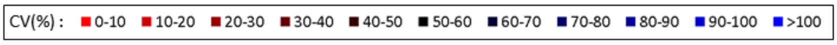

Supplementary Figure S12. The results of cluster aided multivariate curve resolution-alternating least squares (MCR-ALS) (urinary dataset).

Typical concentration profiles and spectral profiles of 21 clusters are shown. Scales of these graphs are provided in arbitrary units. The number in parentheses indicates the cluster size.

Supplementary Figure S13

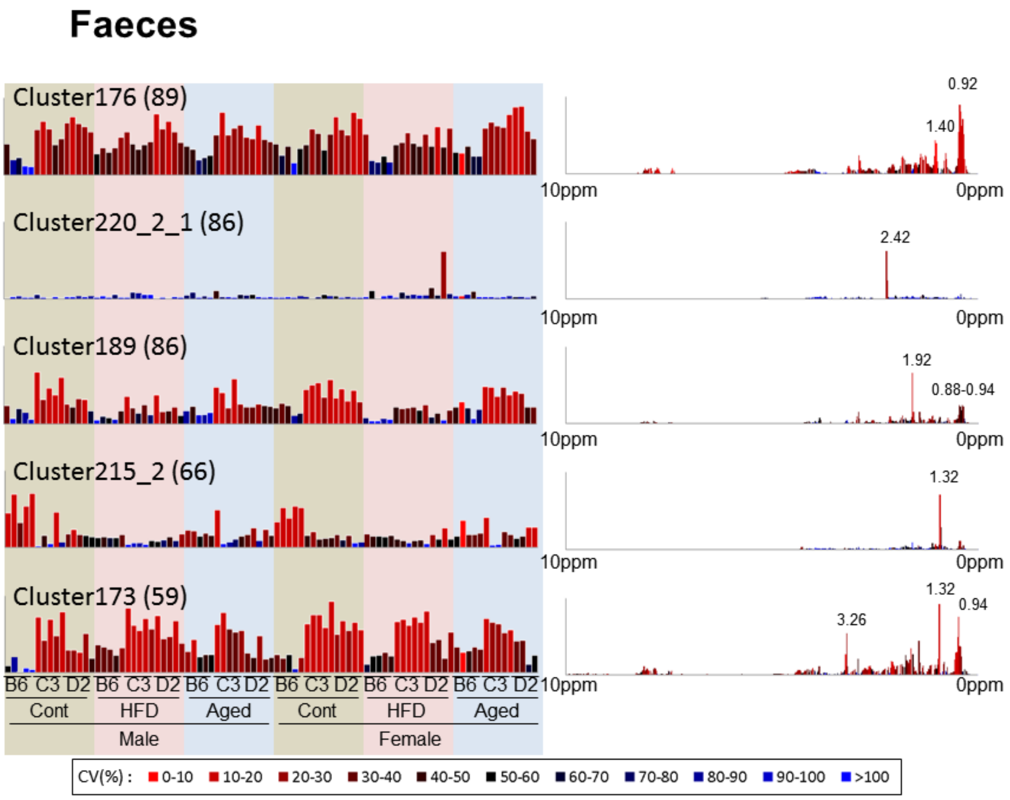

Supplementary Figure S13 (continued)

Faeces

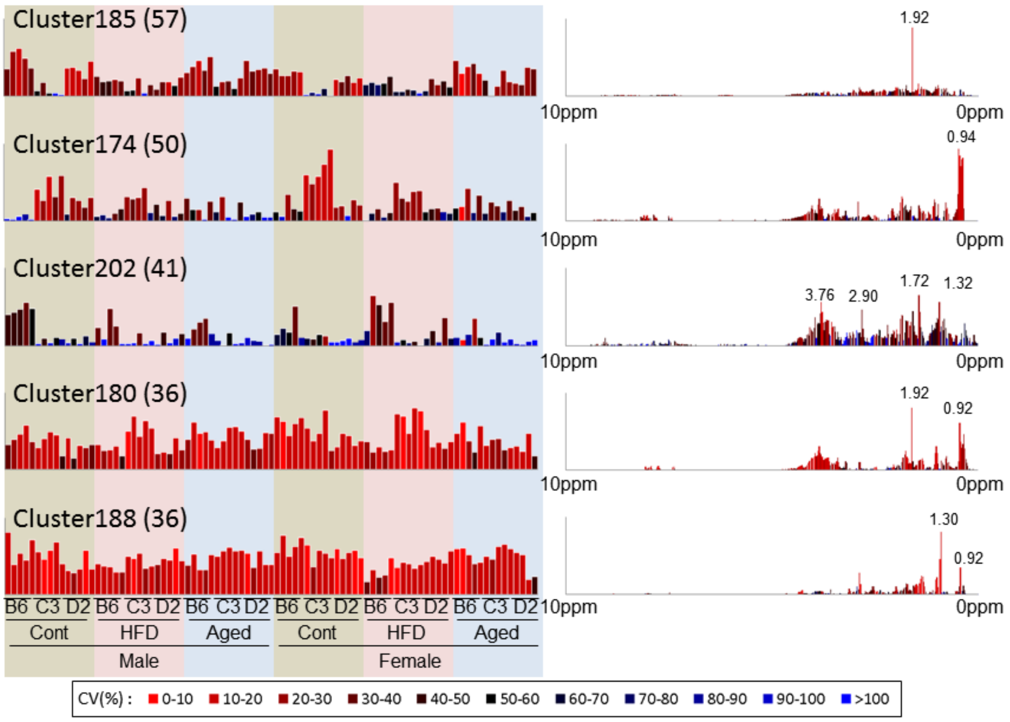

Supplementary Figure S13 (continued)

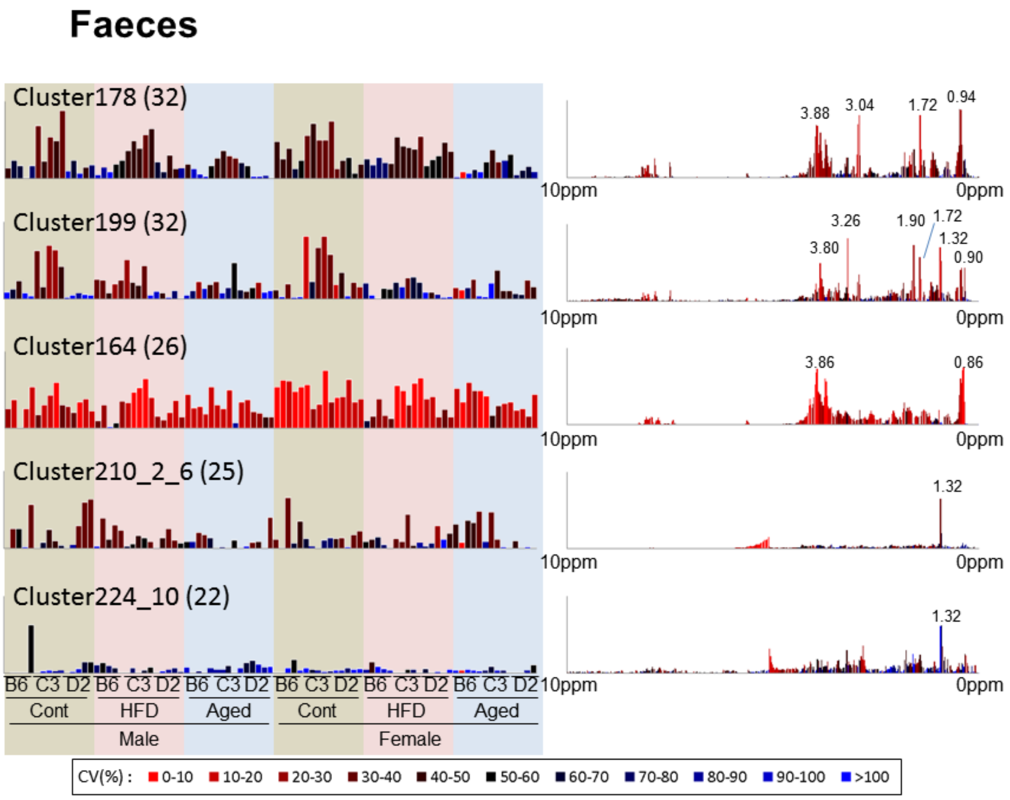

Supplementary Figure S13 (continued)

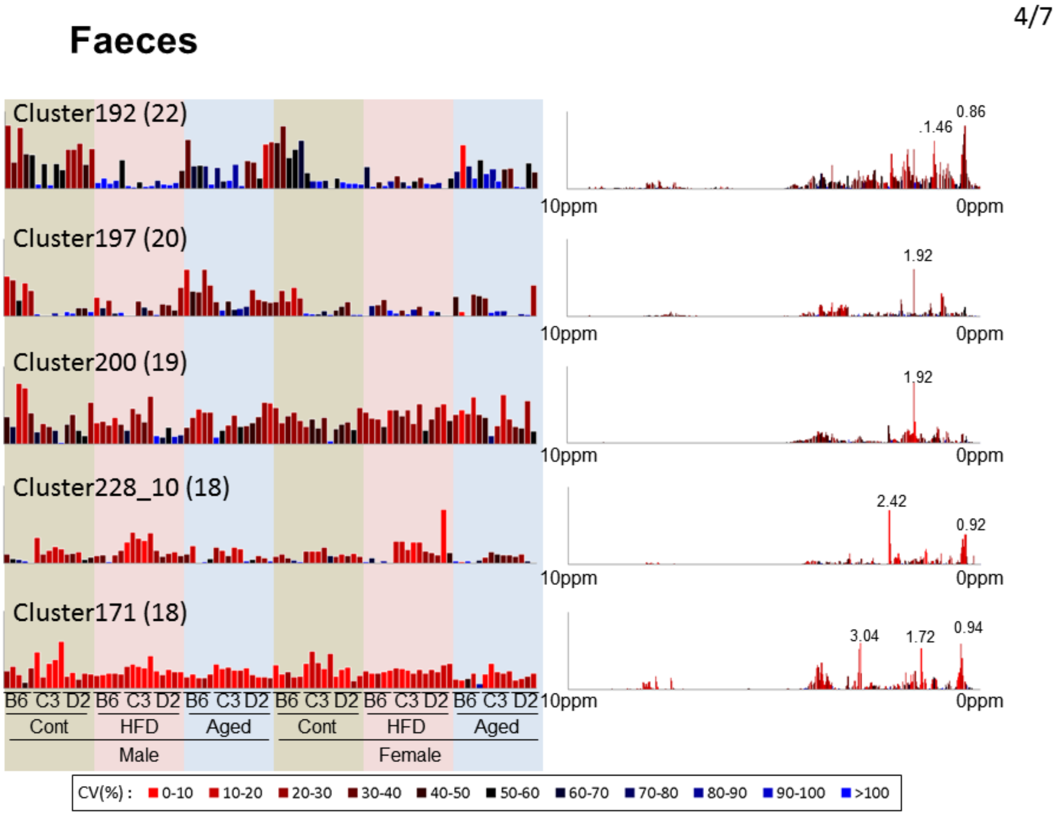

Supplementary Figure S13 (continued)

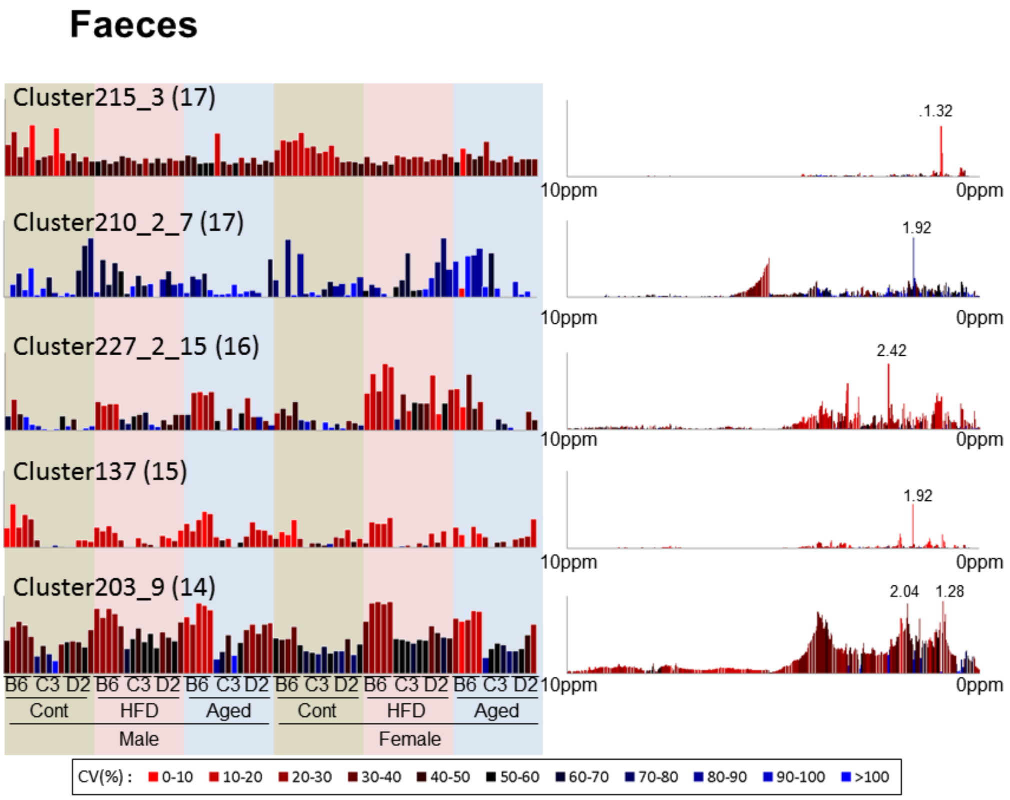

Supplementary Figure S13 (continued)

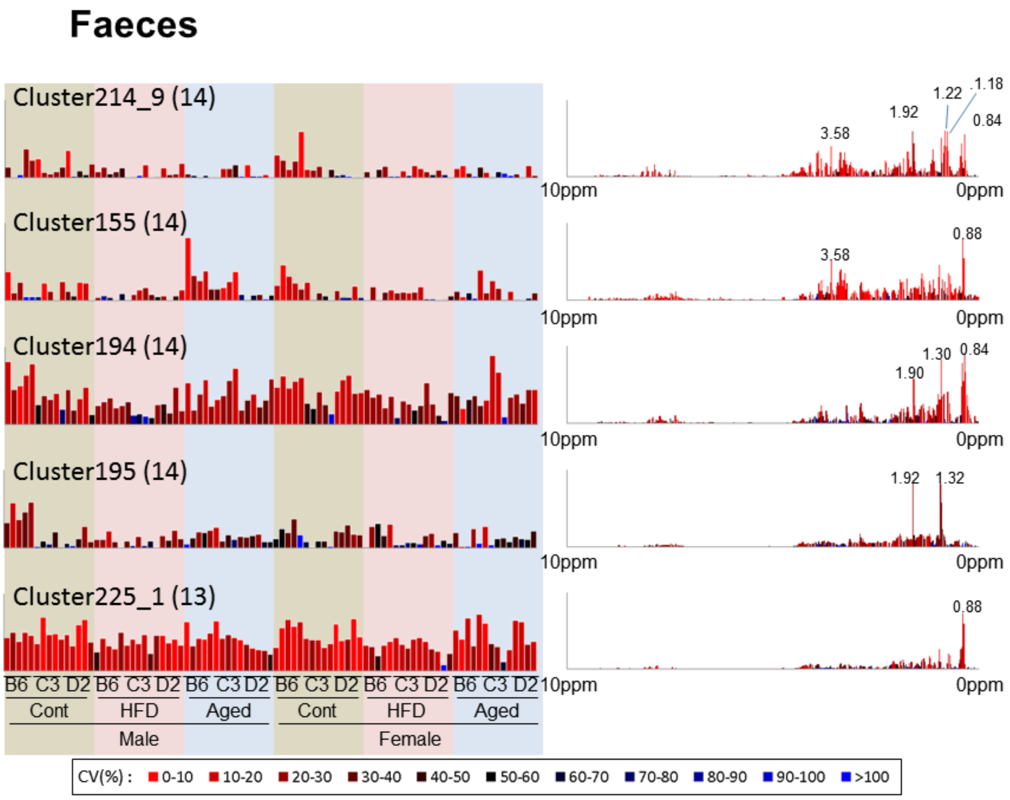

Supplementary Figure S13 (continued)

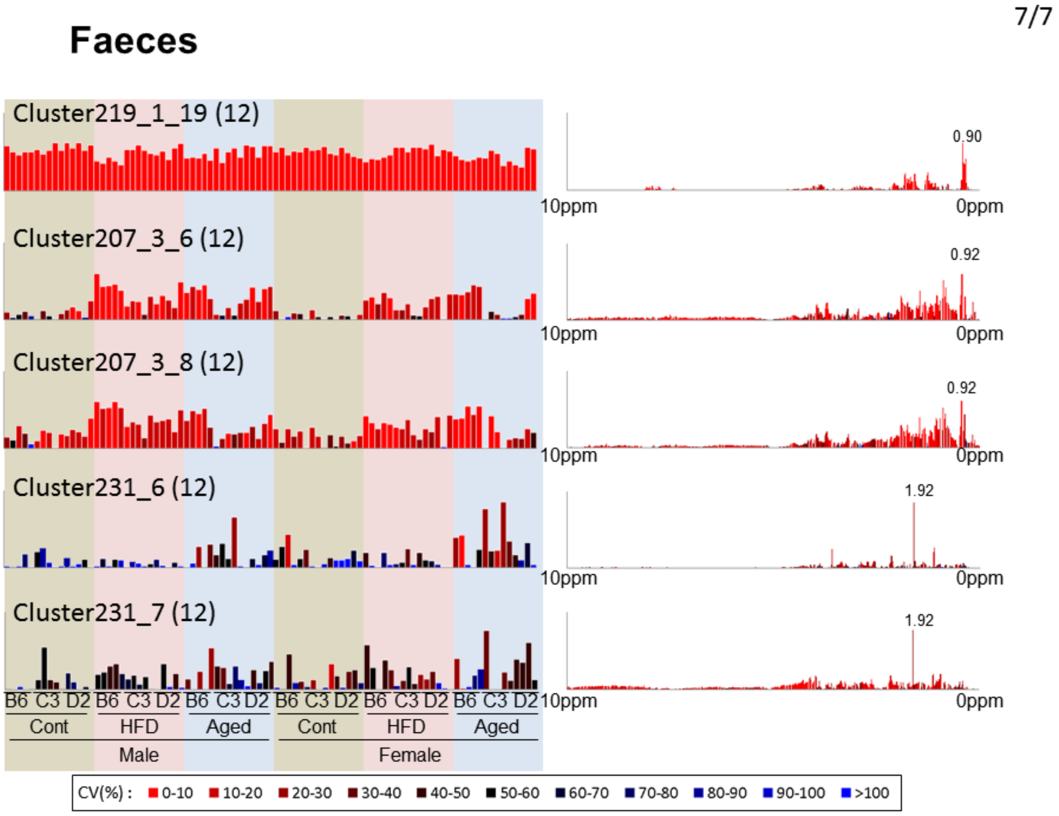

Supplementary Figure S13. The results of cluster aided multivariate curve resolution-alternating least squares (MCR-ALS) (fecal dataset).

Typical concentration profiles and spectral profiles of 35 clusters are shown. Scales of these graphs are provided in arbitrary units. The number in parentheses indicates the cluster size.

Supplementary Figure S14

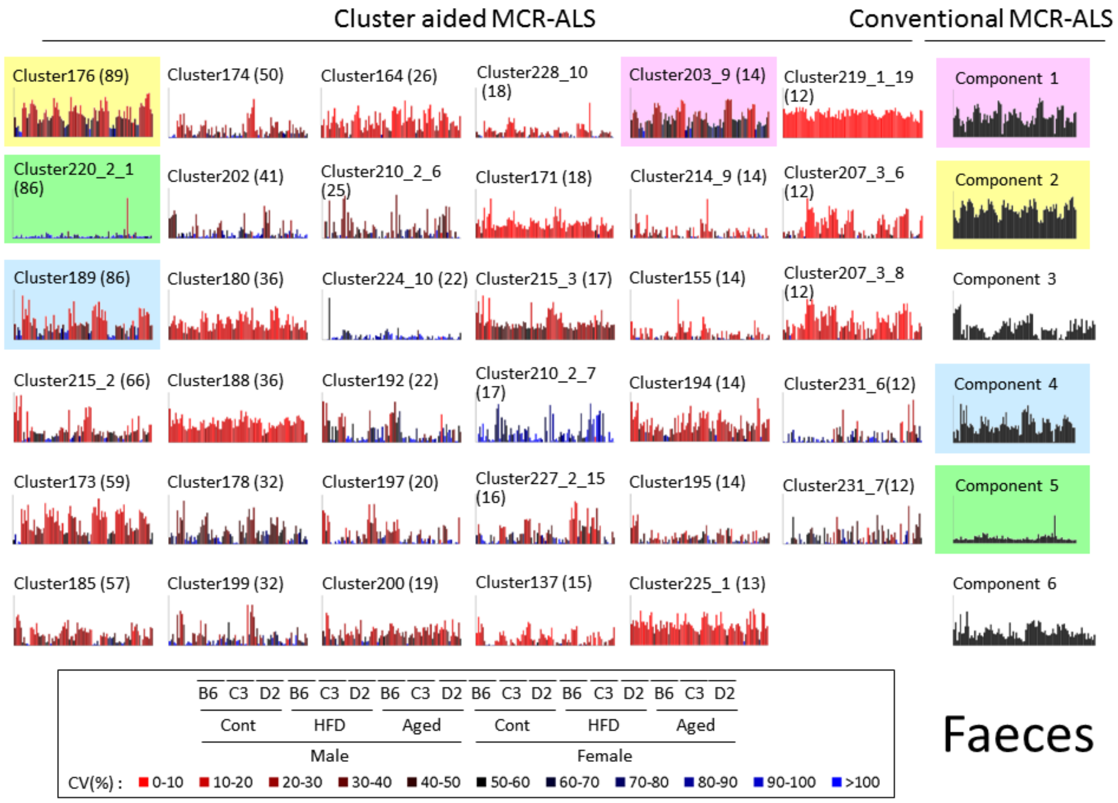

Supplementary Figure S14. The results of cluster aided multivariate curve resolution-alternating least squares (MCR-ALS) and conventional MCR-ALS in fecal data analysis.

Concentration profiles of the results of fecal data analysis. In the bar graph, the order of the samples is indicated at the bottom of the figure. B6: C57BL/6J, C3: C3H/HeJ, D2: DBA/2J, Cont: control group, HFD: high-fat-diet-fed group, Aged: aged group. Typical concentration profiles in 35 identified reliable clusters analyzed by cluster aided MCR-ALS are on the left side of the figure. Six components analyzed by conventional MCR-ALS are on the right side. The number in parentheses indicates the cluster size. Colored clusters/components indicate that the component belongs to the same color cluster. Scales of bar graphs are given in arbitrary units. The colors of the bars correspond to coefficient of variation.

Supplementary Figure S15

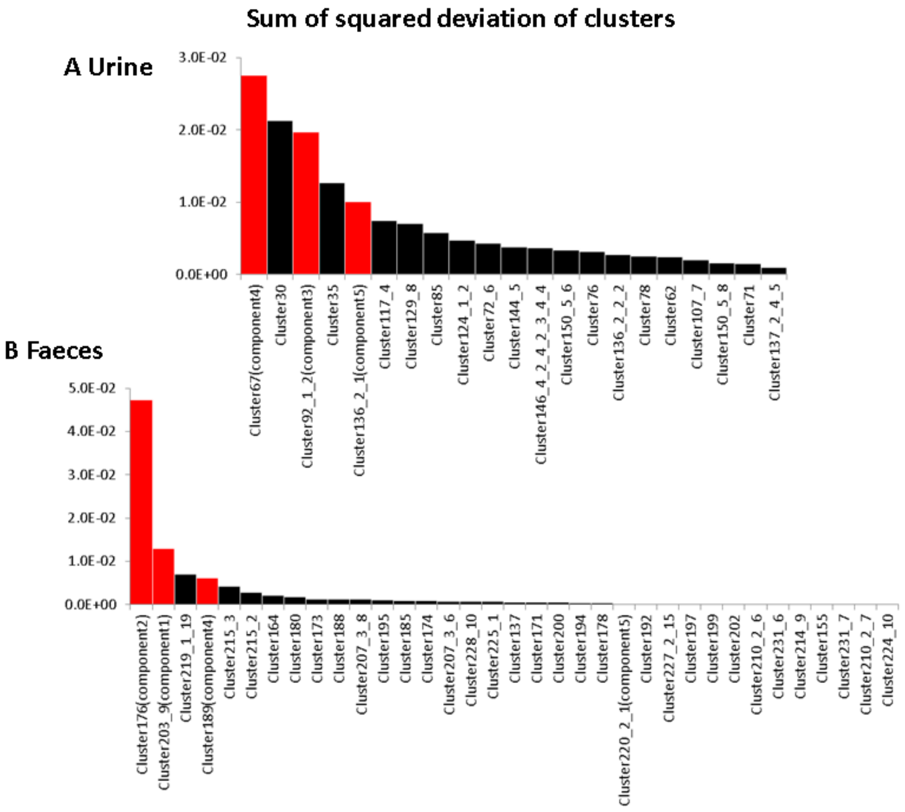

Supplementary Figure S15. Sum of squared deviation of clusters.

The sum of squared deviation of clusters, estimated by cluster aided multivariate curve resolution-alternating least squares (MCR-ALS), is represented by a bar graph. A: Sum of squared deviation of clusters estimated by urinary data analysis. B: Sum of squared deviation of clusters estimated by fecal data analysis. Red bar indicates that the component emerged in both conventional and cluster aided MCR-ALS.
